# Genome-wide analysis of DNA polymorphisms, the methylome and transcriptome revealed that multiple factors are associated with low pollen fertility in autotetraploid rice

**DOI:** 10.1371/journal.pone.0201854

**Published:** 2018-08-06

**Authors:** Xiang Li, Hang Yu, Yamin Jiao, Muhammad Qasim Shahid, Jinwen Wu, Xiangdong Liu

**Affiliations:** 1 State Key Laboratory for Conservation and Utilization of Subtropical Agro-Bioresources, South China Agricultural University, Guangzhou, China; 2 Guangdong Provincial Key Laboratory of Plant Molecular Breeding, South China Agricultural University, Guangzhou, China; 3 College of Agriculture, South China Agricultural University, Guangzhou, China; Wuhan University, CHINA

## Abstract

Autotetraploid rice is a useful germplasm with high biomass production; however, low fertility is the main barrier in commercial utilization. In our previous study, differential expression of meiosis-related miRNAs was found to be involved in the pollen sterility of autotetraploid rice. However, genome-wide DNA variations and methylomes associated with low fertility of autotetraploid rice are still poorly understood. Here, we measured both global DNA variations and the methylome and compared them with the transcriptome during pollen development in autotetraploid rice by high-throughput sequencing. A total of 34416 SNPs, 6993 InDels, 1003 SVs and 25 CNVs were detected, and 11367 and 41117 differentially methylated regions showed hypermethylation and hypomethylation in 02428-4x. In total, 1110 genes displayed differentially expression in 02428-4x during meiosis, of these six harbored CNVs, including four upregulated genes with gain CNVs, such as *LOC_Os11g38620*. We identified 122 genes by comparing with the previous data that might be associated with low fertility during pollen development in 02428-4x. Of the 122 gens, 98 were displayed methylation and differential expression, including *OsMADS98*, *CYP703A3* and *OsABCG26*. The downregulation of these three genes were confirmed by qPCR during meiosis of 02428-4x, which played pivotal roles in pollen fertility. These results indicate that the low fertility of autotetraploid rice is not only caused by the differential expression of genes involved in pollen development, but also by sequence variation and differential methylation, suggesting that the reason for pollen sterility in autotetraploid rice is complex and might be affected by multiple factors.

## Introduction

As important speciation mechanisms, whole-genome duplication (WGD) events are widespread in plant evolution. Autopolyploids show different phenotypes compared to their diploid progenitors, which might be caused by the duplicated genes retaining, losing, or acquiring new functions [1,2]. Though autopolyploidy may be advantageous for some agronomic traits, it also comes with challenges during meiosis due to the increase in chromosome numbers [3,4].

Autotetraploid rice is a useful germplasm resource generated by chromosome doubling; however, autotetraploid rice presents low fertility, which becomes the main bottleneck for its utilization. Partial pollen sterility is one of the main reasons for the low fertility of autotetraploid rice [5]. Cytological observations have revealed that abnormal chromosome behavior and microtubule patterns act synergistically in the pollen sterility of autotetraploid rice [6]. Transcriptome analyses have revealed that irregularity of some pivotal genes, such as *Os11g0146800* (*OsDMC1B*) and *Os09g0506800* (*PAIR2*), may cause abnormal meiosis and lead to low pollen fertility of autotetraploid rice [7]. In addition, differentially expressed patterns of miRNAs, phasiRNAs, and TE-siRNAs during development have been identified in autotetraploid rice, and some meiosis-related miRNAs are involved in low pollen fertility [8,9]. However, genome-wide DNA variations and methylomes associated with low fertility in autotetraploid rice are still poorly understood, and little information is available concerning comprehensive analyses combined with DNA polymorphism, methylome and transcriptome data from the same field.

Next-generation sequencing (NGS) technology represents a powerful tool that can be used to discover abundant DNA polymorphisms, such as single nucleotide polymorphisms (SNPs) and insertion–deletion polymorphisms (InDels), in mutated individuals [10–13]. Recently, Yan et al. [14] reported that rice mutants exhibited abnormal spikelets or male sterility as well as SNPs in important meiosis-related genes. Immunoprecipitation of methylated DNA by monoclonal antibodies specific to 5-methylcytidine sequencing (MeDIP-Seq) can be used to detect methylated DNA in whole genomes and has been utilized in rice, maize and poplar [15–17]. Hypermethylation was identified in PA64S (sterile) rice compared to PA64S (fertile); and 1258 differentially methylated regions (DMRs) were identified between them [16].

To date, combinations of multiomic techniques have greatly promoted research in plants such as *Paulownia* [18] and rice [19] and even in polyploids [20,21]. Asymmetrical changes in small RNAs, transposons and gene expression between two resynthesized wheat allotetraploids can explain the evolution of wheat allotetraploids [21]. Zhang et al. [22] found that the increasing methylation of class II transposable elements (TEs) in autotetraploid rice could suppress the expression level of nearby genes in response to genome dosage effects. In addition, by using a multiomic analysis, Wang et al. [23] reported that rice interploidy crosses could disrupt gene expression, epigenetic regulation, and even seed development. Hence, integrating multiomic results are useful in polyploid plant studies.

Although information on the cytogenetics and transcriptomes of autotetraploid rice is available [7,9,24], no study has addressed how multiple factors affect pollen fertility in autotetraploid rice. Here, an autotetraploid rice, 02428-4x, and its diploid counterpart (02428-2x) were used for resequencing via NGS to detect DNA methylation by MeDIP-Seq, to analyze the differentially expressed genes by RNA sequencing, and then to reveal the male sterility-related genes. Finally, comprehensive analyses of DNA polymorphisms, the methylome and the transcriptome associated with sterility were carried out to identify the factors involved in the low pollen fertility of autotetraploid rice.

## Materials and methods

### Rice material

Autotetraploid rice, 02428-4x, was obtained from the chromosome doubling of 02428-2x (*Oryza sativa* L. subsp. *japonica*) by colchicine and was self-crossed for more than 27 generations in our lab [9]. The plants were planted in an experimental field at South China Agricultural University. The leaves of two-week-old seedlings of 02428-4x and 02428-2x were collected for resequencing, and the genomic DNA was extracted by using the modified cetyltrimethyl ammonium bromide (CTAB) method. Anthers at pollen meiosis stage were collected from both lines and stored at -80°C for transcriptome sequencing and methyl-DNA immunoprecipitation sequencing (MeDIP-Seq). Anther at the premeiotic interphase and single microspore stage were collected for digital gene expression profiling. Total RNA was isolated from the anthers using a TRK1001 total RNA purification kit (LC Science, Houston, TX, USA) following the manufacturer’s procedure. The DNA of the anthers was also extracted by the CTAB method.

### Semi-thin sections

Florets of 02428-4x and 02428-2x were collected and fixed in FAA solution (50% ethanol:acetic acid:methyl aldehyde = 89:6:5) for 48 hours, rinsed several times with 50% ethanol and dissected to remove the anthers. The anthers were dehydrated in a series of ethanol solutions (70%, 80%, 90% and 95% ethanol) for 30 min each. The dehydrated anthers were then embedded by a Leica 7022 historesin embedding kit (Leica, Nussloch, Germany) following the manufacturer’s recommended protocol and polymerized at normal temperature. The transverse section of the anthers was sectioned by a Leica RM2235 manual rotary microtome with a thickness of 3 μm. Finally, the sections were stained with 1% toluidine blue O and sealed with neutral balsam, after which they were imaged with a Motic BA310 system.

### Genomic resequencing

The genomic DNA of leaves was randomly fragmented by sonication, and the desired lengths of the DNA fragments were gel purified. The DNA fragments were ligated to adapters for library construction. Finally, resequencing was performed using an Illumina HiSeq 2500 platform (Biomarker, Beijing, China). The raw data with adapter sequences and low-quality reads (base quality value less than 20) were filtered using FastQC software. After filtration, the clean data were aligned to the Nipponbare reference genome (MSU V7.0) by Burrows-Wheeler Aligner (BWA) software [12]. Single nucleotide polymorphisms (SNPs) and insertions and deletions (InDels) were detected by GATK software and then annotated using SnpEff. The structural variations (SVs) and copy number variations (CNVs) were analyzed by BreakDancer [25] and FREEC [26], respectively; those with a read depth less than 10 were removed.

### Transcriptome sequencing

The quantity and purity of the total RNA were analyzed with a Bioanalyzer 2100 and RNA 6000 Nano LabChip Kit (Agilent, Palo Alto, CA, USA) with a RIN number > 8. Approximately 10 μg of total RNA (anthers in meiosis) was subjected to isolate poly (A) mRNA with poly-T oligo attached magnetic beads. Following purification, the mRNA was fragmented into small pieces using divalent cations under elevated temperature. The cleaved RNA fragments were then reverse-transcribed to construct a final cDNA library according to the manufacturer’s instructions of the mRNA-Seq sample preparation kit (Illumina, San Diego, CA, USA). Finally, we performed paired-end sequencing with an average insert size of 300 bp (±50 bp) on an Illumina HiSeq 2000 platform (LC Sciences, Hangzhou, China) following the manufacturer’s recommended protocol. After sequencing, the low-quality reads, reads containing sequencing adaptors, reads containing sequencing primers, and reads containing nucleotides with quality scores lower than 20 (Q<20) were removed. The clean reads were subsequently aligned to the *Oryza sativa* genome (MSU V7.0) using the TopHat package. The aligned reads were then used to assemble transcripts of each sample independently using the Cufflinks program [27]. The aligned reads were further processed by Cufflinks, which uses the normalized RNA-Seq fragment counts to measure the transcript relative abundances. The measurement unit of fragments is per kilobase of transcript per million fragments (FPKM). Genes with *P*-values < 0.05 and |log2 (fold change ratio)| >1 were considered differentially expressed genes.

The total RNA (~10 μg) of the anther in the premeiotic interphase and single microspore stage were used to generate cDNA according to the Illumina protocol (Illumina, San Diego, CA, USA) for digital gene expression profiling. The products were single-end sequenced on an Illumina HiSeq 2000 (LC Sciences, Hangzhou, China) following the vendor’s recommended protocol. After the raw reads filtration, the clean reads mapped to the *Oryza sativa* genome (MSU V7.0) using Bowtie, with only one base pair mismatch allowed [28]. The expression of candidate genes was normalized as reads per kilobase of exon model per million mapped reads (RPKM). Genes with *P*-values < 0.05 and |log2 (fold change ratio)| >1 for the comparison samples (02428-2x vs 02428-4x) were considered differentially expressed.

### Methyl-DNA immunoprecipitation sequencing

Before carrying out MeDIP-Seq, we followed the method described by Sati et al. [29]. First, we sonicated anther DNA to produce random fragments ranging in size from 150 to 800 bp using an AIR™ DNA Fragmentation Kit (Bioo Scientific, Austin, Texas, USA). Based on the Illumina manufacturer’s recommended protocol, we then end-repaired, phosphorylated and A-tailed the fragmented DNA and ligated the adapters. The adaptor-ligated DNA was subsequently subjected to MeDIP enrichment using a MethylMiner™ Methylated DNA Enrichment Kit (Life Technologies, New York, USA). The efficiency of enrichment was checked by real-time quantitative PCR. Then, PCR amplification was performed, and target bands were excised from the gel to produce libraries; these libraries were quantified using an Agilent 2100 analyzer. Finally, MeDIP-Seq was performed on an Illumina HiSeq 2000 platform (LC Sciences, USA). The raw data of low-quality reads were filtered after sequencing, and clean data were mapped and assembled onto a reference genome (MSU V7.0). The CpG enrichment (CpG coverage ≥ 5X) and differentially methylated region (DMRs with a *P*-value < 0.01 and |log2 (fold change ratio)| >1) analysis were performed using the MEDIPS software package [30]. RPKM (reads per kilobase of exon model per million mapped reads) was used to measure the reads. The CpG islands (CGIs) followed the three basic characteristics described by Sati et al. [29]: length greater than 200 bp, GC content > 50% and observed/expected CpG > 0.6. Different types of DMRs were classified based on gene structure (promoter, exon, intron and intergenic regions), repetitive elements (BLASTed to RepeatMasker) and CGIs and CGI shores (~2 kb near the CpG islands).

### Functional analysis of the genes

Gene Ontology (GO) analysis of the genes was performed using AgriGO2 [31]. Significance was expressed when the *P*-value < 0.05, and protein-protein interaction networks were determined using STRING [32].

### Verification analysis

PCR was used to amplify the polymorphic loci in a 20 μL volume containing 30 ng of DNA template, 0.15 μmol/L primer pairs, 1.0 μL of dNTPs (2.0 mmol/L each), 1 U of Taq polymerase, and 1× PCR buffer (50 mmol/L KCl, 10 mmol/L Tris-HCl [pH 8.3], 1.5 mmol/L MgCl2, 0.01% gluten). The PCR was done according to Liu et al. [12]. The PCR products were examined by agarose gel electrophoresis and sequenced by Sanger sequencing. The Sanger sequencing results were assembled by DNAMAN software and further aligned to the reference genome sequences to validate the variations in polymorphic loci using ClustalW software. The total RNA obtained from rice anthers was reverse-transcribed using a PrimeScript™ RT reagent kit (Takara, Otsu, Shiga, Japan). Real-time PCR was performed using SsoAdvanced Universal SYBR Green Supermix (Bio-RAD, Hercules, CA, USA) for amplification of the PCR products. Real-time PCR conditions were conducted following the instructions of the manufacturer (Bio-RAD). Ubiquitin was used as an internal reference. All qRT-PCR amplifications were carried out in triplicate, and the results are presented as the means ± standard deviations. The relative expression of genes was calculated by the 2^–ΔΔCT^ method [33]. All the primers used for PCR and qRT-PCR were developed by Primer 5.0 software, checked by NCBI Primer-BLAST, and are listed in S1 and S2 Tables.

## Results

### The anther development in autotetraploid rice as observed by semi-thin sections

**Fig 1:**
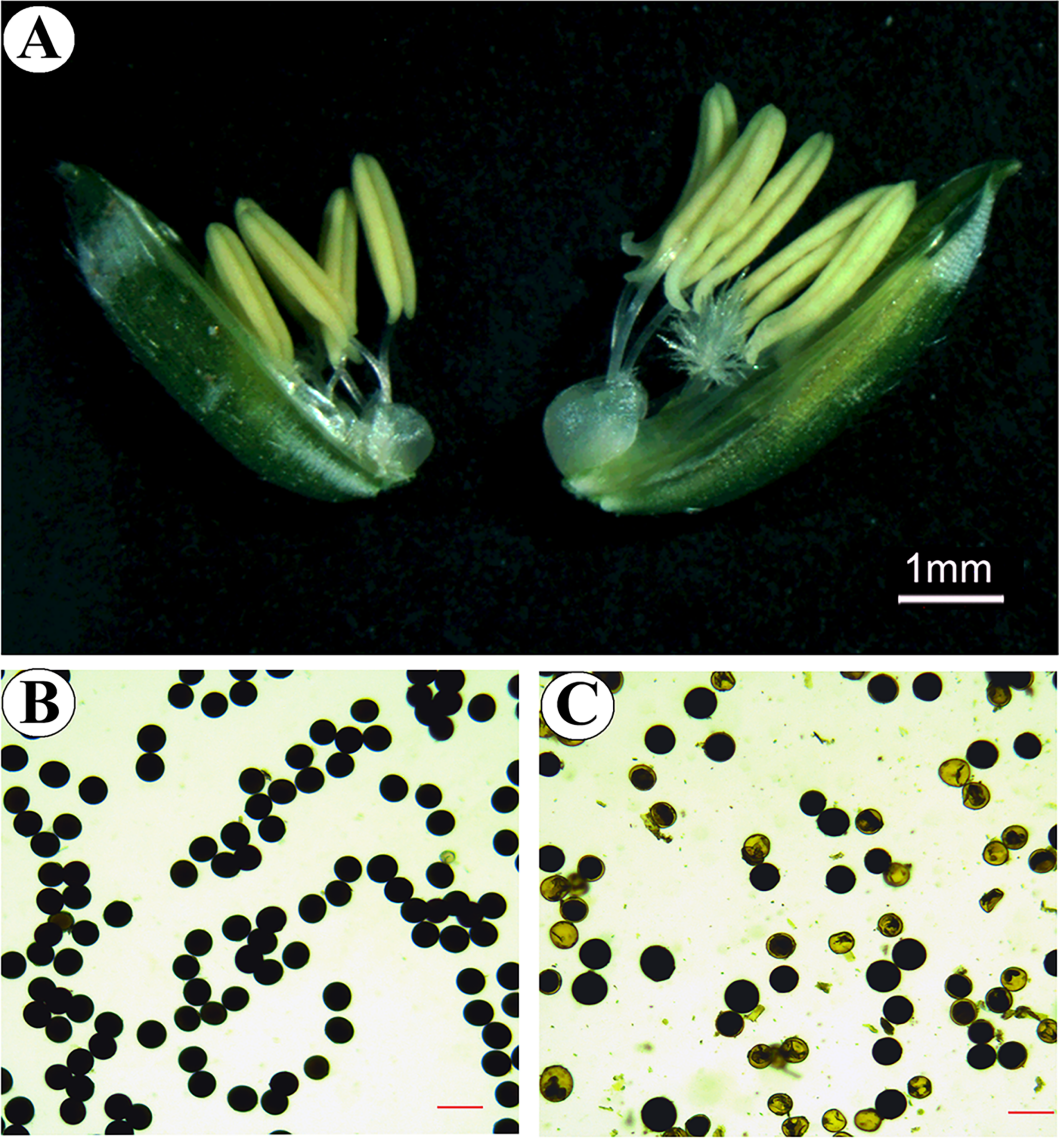
Cytological observations of pollen morphology. (A) Anther phenotypes of diploid (left) and autotetraploid (right) rice. (B and C) 1% I_2_-KI staining of the pollen grains of diploid and autotetraploid rice. Bar = 200 μm.

**Fig 2:**
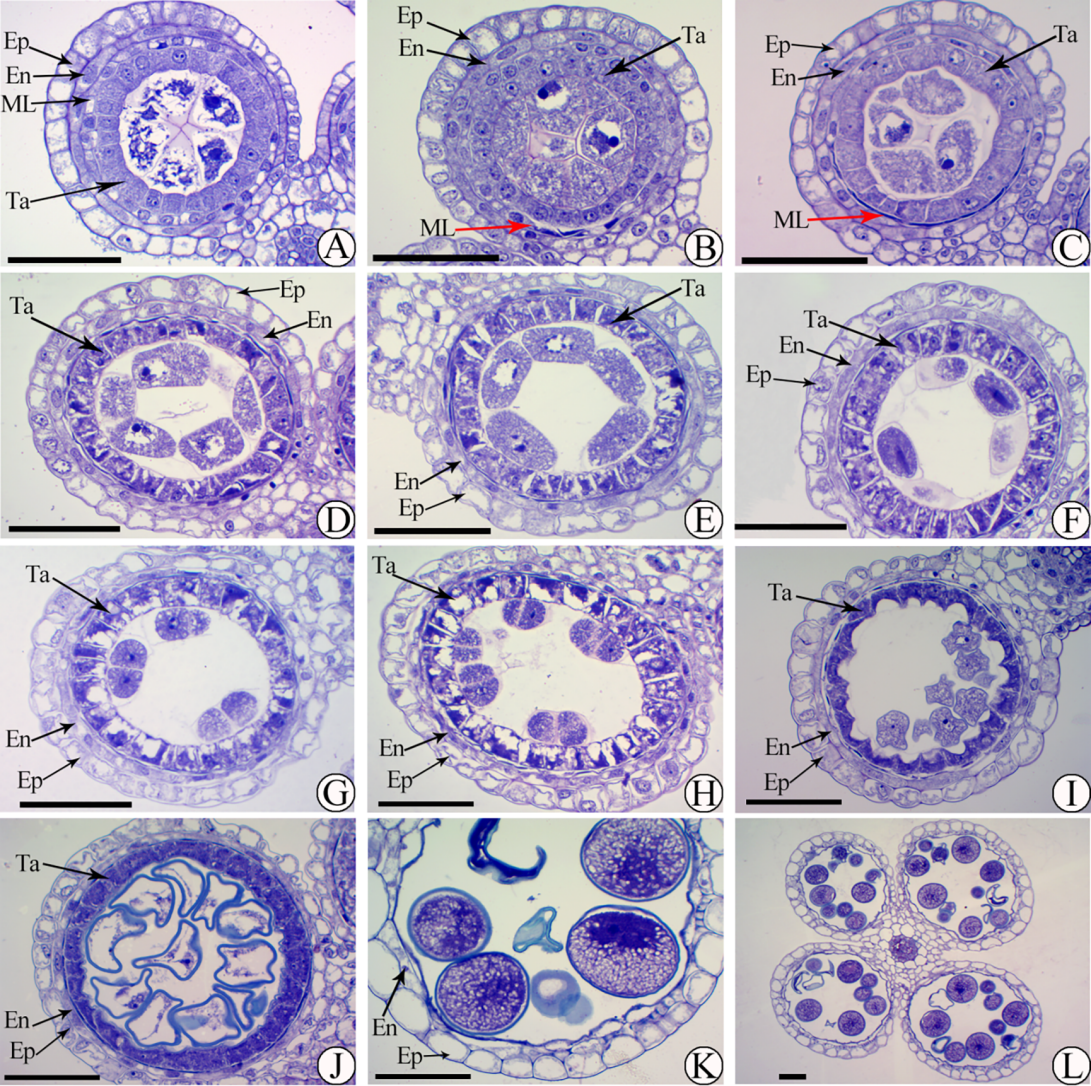
Semi-thin sections showing anther development in 02428-4x. (A) Premeiotic interphase, (B-H) meiosis stage, (I-J) single microspore stage, (K, L) mature pollen. Ep, En, ML and Ta indicate the epidermis, endothecium, middle layer and tapetum, respectively. The red arrow indicates the degenerated middle layer. Bars = 50 μm.

**Fig 3:**
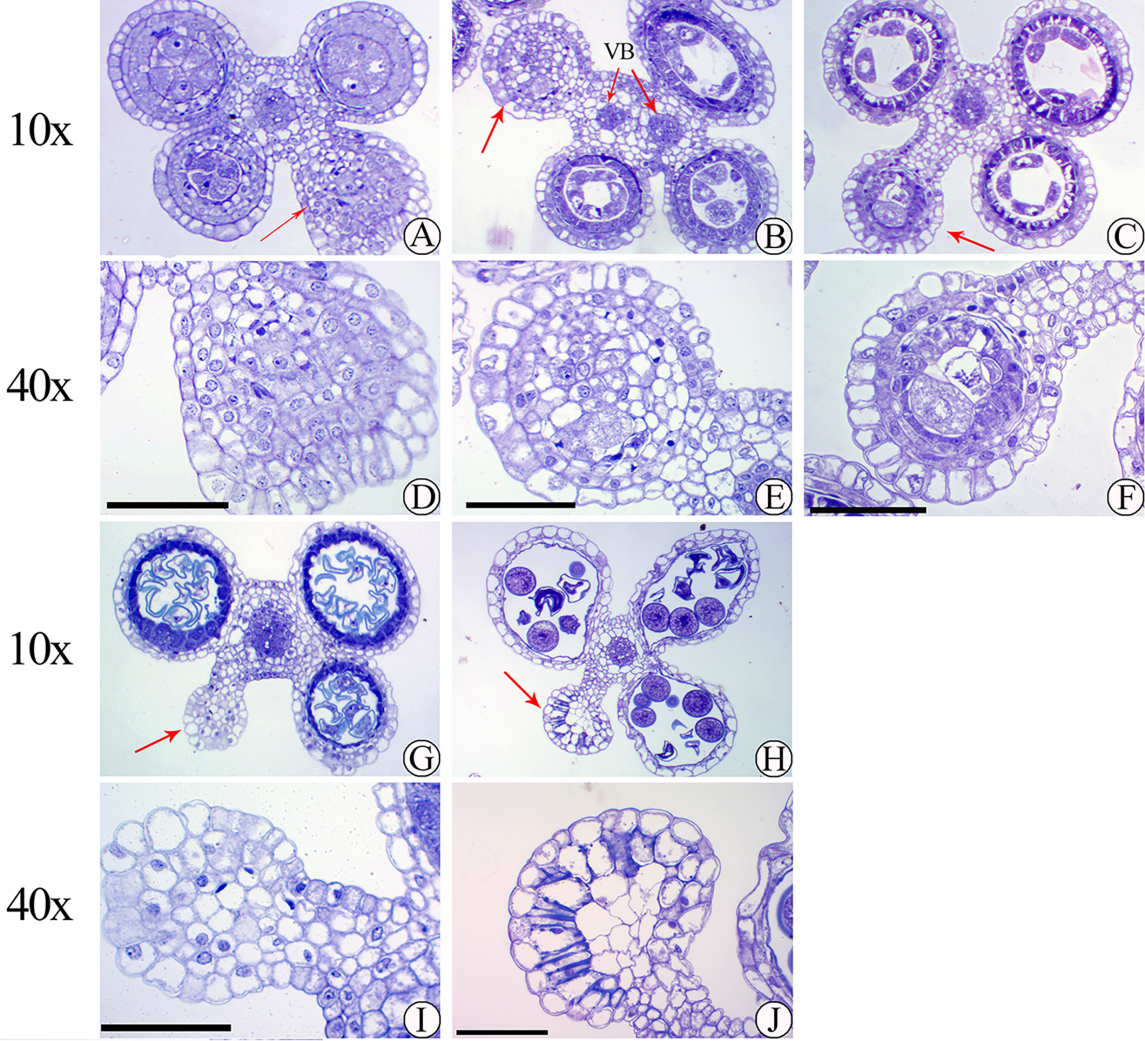
Abnormal anther locule during anther development of autotetraploid rice. (A) Premeiotic interphase, (B-C) meiosis stage, (G) single microspore stage, (H) mature pollen, (B) double vascular bundle (VB). (D, E, F, I, J) abnormal anther locule of each stage. The red arrow indicates the abnormal anther locule. Bars = 50 μm.

**Fig 4:**
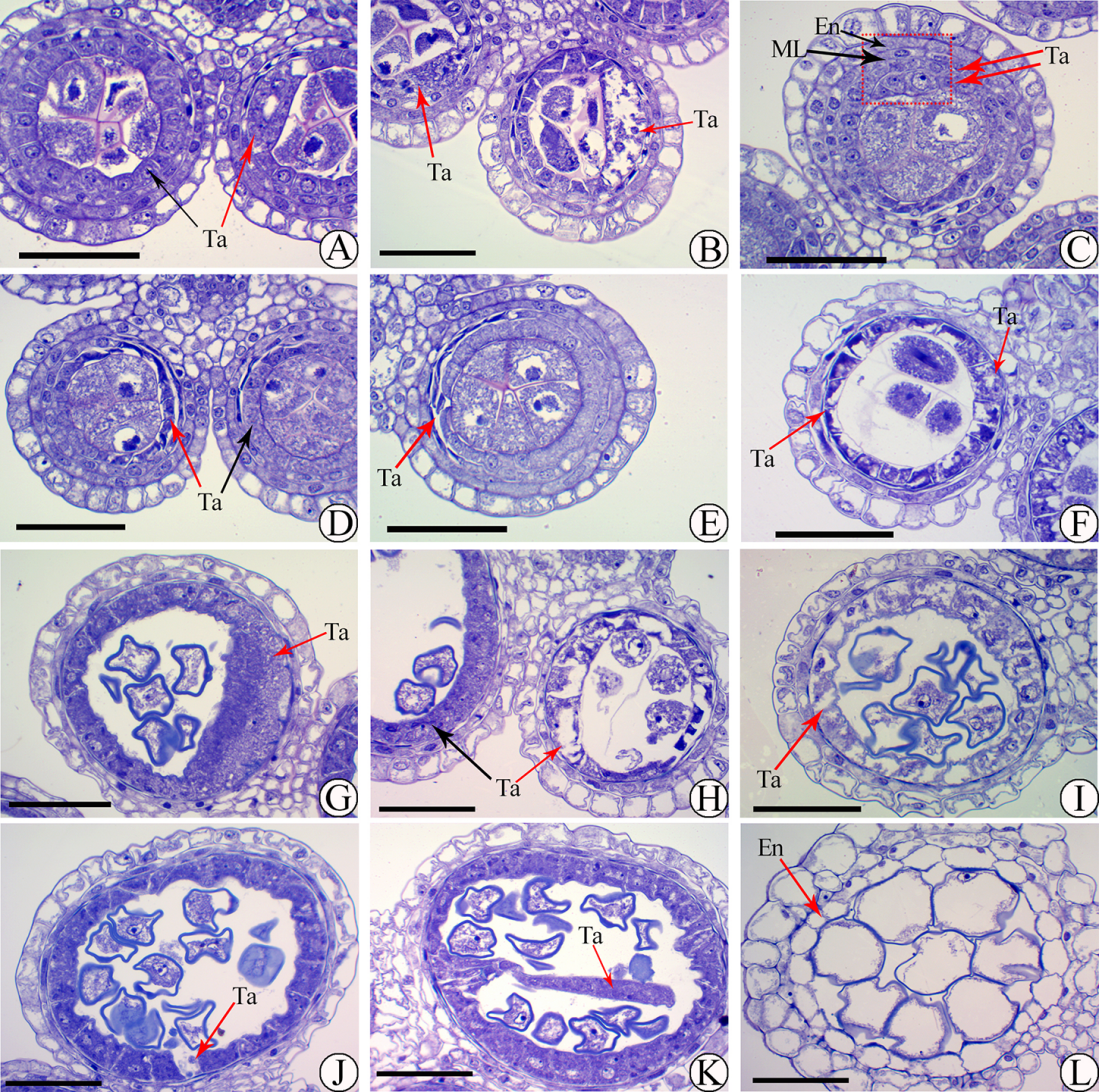
Abnormal tapetum during anther development in autotetraploid rice. (A, B) Giant tapetal cells (A) and prevacuolation of giant tapetal cells (B) during premeiotic interphase. (C-E) Redundant tapetal cells (C) and tapetum degenerating along with the middle layer (D, E) during meiosis. (F, G) Unbalanced tapetum size after meiosis. (H, I) Tapetum cytoplasm that is not deeply stained at the microspore stage. (J, K) Degeneration of the tapetum (J) and abnormal proliferation (K) during the microspore stage. (L) Arrested microspores and larger endothecium in the mature pollen stage. En, ML and Ta indicate the endothecium, middle layer and tapetum, respectively. The red arrow indicates the abnormalities. Bars = 50 μm.

Low pollen fertility was found in 02428-4x compared to 02428-2x (Fig 1). The development of the anthers of 02428-4x (Fig 2) was similar to that of 02428-2x (S1 Fig). The anther development could be divided into eight different stages: pollen mother cell formation (Fig 2A), pollen mother cell meiosis (Fig 2B–2H), the early microspore stage (Fig 2I), the middle microspore stage, the late microspore stage, the early bicellular pollen stage (Fig 2J), the late bicellular pollen stage and mature pollen (Fig 2K). However, many abnormalities (~36%) were observed in 02428-4x during anther development (S3 Table). An abnormal anther locule (~6%) with one degenerating locule and a double vascular bundle (Fig 3) was found. In addition, abnormal tapeta (~30%) were found, including giant tapetal cells (Fig 4A) and giant tapetal cell prevacuolation (Fig 4B) during premeiotic interphase; redundant tapetal cells (Fig 4C) and thinned/degenerate tapeta along the middle layer (Fig 4D and 4E) during meiosis; unbalanced tapetal size after meiosis (Fig 4F and 4G); and tapetal cytoplasm with no concentration (Fig 4H and 4I), degenerated tapetum (Fig 4J) and abnormal tapetal proliferation (Fig 4K) during the microspore stages. Moreover, arrested microspores and larger endothecia were found in the mature pollen stage (Fig 4L).

### Detection and distribution of DNA variations in autotetraploid rice

In total, 113 million and 111 million clean reads were obtained in 02428-2x and 02428-4x by resequencing, respectively (S4 Table). Approximately 96% of the clean reads were mapped to the Nipponbare reference genome (MSU v7.0) by BWA software, and the average depth and coverage were more than 36x and 94%, respectively (S4 Table), which suggested that high quality clean reads could be used for further analysis.

Different types of variations were identified in 02428-2x and 02428-4x when compared to the reference genome (S2 Fig). A total of 459416 and 459327 SNPs, and 117296 and 117278 InDels were identified in 02428-2x and 02428-4x, respectively (S5 Table). Chromosomes 8 and 11 exhibited the greatest SNP rate (1 change for every 379 bases and 520 bases, respectively) between 02428-2x and the reference genome; same results were observed in 02428-4x (S3 Fig). A similar distribution of the InDel rate was detected in both 02428-2x and 02428-4x (S3 Fig). Approximately 70% of the SNP base substitutions were transitions in 02428-2x and 02428-4x; this percentage was greater than that for the transversions (S3 Fig). The most frequent length of insertions and deletions was 1 bp, and 71.80% and 71.84% of InDels had a length of 1–3 bp in 02428-2x and 02428-4x, respectively (S3 Fig). Seventy-five polymorphic loci related to 21 genes were selected for PCR verification (S1 Table). The PCR results showed that the variations in the sequence were consistent with the resequencing data, which demonstrated the accuracy of the samples used in this study. In addition, the resequencing results were generally similar to previous resequencing results of the materials in 2013, indicating that the materials were stable.

To further investigate the variants in 02428-4x, we compared the variants to 02428-2x at depths ≥5 and ≤ 100. A total of 41409 polymorphic loci, including 34416 SNPs and 6993 InDels, were identified between 02428-4x and 02428-2x (S4 Fig). The densities of SNPs and InDels were 9.31 and 1.85, respectively, at 100 kb (S6 Table). According to the position and annotation of the reference genome, 62.71% of the polymorphic loci were classified in intergenic, downstream and upstream regions, while only a few (18.75%) were located in exons. The number of nonsynonymous coding SNPs was 3645; 174 frame shift InDels and 76 codon insertion/deletion InDels were also detected (S7 Table). Most of the polymorphic loci were heterozygous, and only 185 variations (0.45%) were homozygous, including 78 SNPs and 107 InDels (S8 Table). Of these homozygous and heterozygous variations, a total of 1303 genes were associated with missense mutations, whereas 202 genes were related to major impact mutations (such as frame shifts and nonsense mutations) (S8 Table). Finally, eight genes associated with homozygous polymorphisms between 02428-4x and 02428-2x were documented (Table 1), including *LOC_Os03g06260*, *LOC_Os05g10440*, *LOC_Os08g24060* and *LOC_Os11 g36880*, which are associated with major impact mutations, and *LOC_Os01g37780*, *LOC_Os09g37270*, *LOC_Os11g27670* and *LOC_Os11g35756*, which are associated with missense mutations. Of these genes, six were related to transposable elements, one was annotated as an expressed protein (*LOC_Os01g37780*) and one was *LOC_Os09g37270* (*OsRacGEF1*, ATROPGEF7/ROPGEF7). No Gene Ontology (GO) enrichment results were found for the eight genes. Promoter region mutations may increase or decrease the gene expression levels [34–36]. We focus on the upstream region that within 2000 bp, and found 45 genes associated with the homozygous variations between 02428-4x and 02428-2x (S8 Table).

**Table 1:** The homozygous DNA polymorphisms between autotetraploid (02428-4x) and diploid (02428-2x) rice.

Gene name	Position	Reference	02428-2x	02428-4x	Amino Acid	Effect	Gene annotation
*LOC_Os01g37780*	21135320	G	G	A	4x (109: P→L)	non-synonymous coding	expressed protein
*LOC_Os03g06260*	3139644	G	G	A	4x (1028: W→*)	Stop gained	retrotransposon protein, putative, unclassified, expressed
*LOC_Os09g37270*	21528934	C	C	T	4x (183: R→H)	non-synonymous coding	ATROPGEF7/ROPGEF7, putative, expressed
*LOC_Os11g27670*	15932019	C	T	C	4x (218: S→G)	non-synonymous coding	retrotransposon, putative, centromere-specific
*LOC_Os11g35756*	20989688	G	G	A	4x (165: C→Y)	non-synonymous coding	transposon protein, putative, CACTA, En/Spm sub-class
*LOC_Os05g10440*	5685907	C	CGGCTTGGCGCGCTT	CGGCTTGGCGCGCTTCAGCT CCCGGAACCAGGCGCAGACA GGCTCGTTGAACTGAGCGAG GAT	2x (640: W→*SAPS?), 4x (640: W→*SSLSSTSLSAPGSGS*SAPS?)	Stop gained/ Frame shift	transposon protein, putative, CACTA, En/Spm sub-class, expressed
*LOC_Os08g24060*	14546675	GTC	GACTC	GACTTGCATTTTGTTC	2x (387: V→D?), 4x (387: V→DLHFV?)	Frame shift	retrotransposon protein, putative, unclassified
*LOC_Os11g36880*	21758504	A	ACGGAGTTCAACAGAGCACT ATCAATTGCACGACCATTAA ATCCTGCCACCCATAACATT TCATATGGAGTATCCCT	ACGGAGTTCAACAGAGCACT ATCAATTGCACGACCATTAA ATCCTGCCACCCATAAC	2x (424: I→IGILHMKCYGWQDLMVVQLIVLC*TP?), 4x (424: I→MLWVAGFNGRAIDSALLNS?)	Frame shift	retrotransposon protein, putative, unclassified, expressed

4x (109: P→L) indicated the number of 109aa change from P to L in autotetraploid rice.

‘*’ indicated the stop codon. ‘?’ indicated the amino acid based on the following nucleotide.

In addition, 2974 and 2868 structural variations (SVs) were found in 02428-2x and 02428-4x after threshold selection (20≤read numbers≤100). The Venn diagram results revealed 91 SVs that were similar between 02428-2x and 02428-4x, whereas 2883 and 2777 SVs were specifically detected in 02428-2x and 02428-4x, respectively (S5 Fig). In total, 1033 and 1003 SVs were located in exons of 02428-2x and 02428-4x, and 1110 genes were associated with these variations. Moreover, a total of 43 regions showed differential copy number variations (CNVs) in 02428-4x compared to 02428-2x, whereas 18 and 25 CNVs were specifically related to 02428-2x and 02428-4x, respectively (S5 Fig). A total of 965 genes were associated with these differential CNVs between 02428-4x and 02428-2x. Furthermore, we found 1386, 978 and 853 genes specifically related to SNPs and InDels, SVs and CNVs by the Venn diagram analysis, respectively (S6 Fig; S9 Table).

Low pollen fertility, abnormal tapeta and abnormal chromosome behaviors were observed in 02428-4x. Therefore, we focused on the polymorphic genes that may be associated with pollen development compared with pollen/meiosis/tapetum genes or meiosis stage-specific genes reported in rice, polyploid rice and *Arabidopsis* [7,37–41]; 127 genes were ultimately detected (S10 Table). Of these 127 genes, 7 were harbored homozygous SNPs/InDels in promoter, including *LOC_Os05g46390* (OsFBX175—F-box domain containing protein) and *LOC_Os12g12260* (diacylglycerol kinase 1). Notably, one meiosis gene, *LOC_Os03g44760* (*OsAM1*, SWI1), which carries SV, was found. Two tapetum genes, including *LOC_Os05g47446* (*OsPDCD5*, expressed protein), which have CNVs, and *LOC_Os10g03660* (*OsADF*, F-box domain-containing protein), which carries heterozygous SNPs/InDels, was also detected. In addition, 45 genes, including 22 and 23 associated with anthers and ovaries, were detected in high fertility neo-tetraploid rice, respectively. A total of 105 polymorphic genes showed a relationship with rice megagametogenesis: 23 were involved in embryo sac mother cell (EMC) premeiotic interphase, 34 were related to the EMC meiosis process, and 48 were associated with nuclear proliferation and maturation of embryo sacs [42,43]. One of these 105 genes, *LOC_Os09g37270* (*OsRacGEF1*), carries homozygous SNPs that cause missense mutation.

### Differentially expressed genes (DEGs) during meiosis in autotetraploid rice

More than 6 million clean data of the meiosis anthers were obtained by transcriptome sequencing (S11 Table). A total of 1110 genes displayed differential expression during PMC meiosis between 02428-4x and 02428-2x. Moreover, 1729 and 2476 differentially expressed genes (DEGs) were detected in the premeiotic interphase (PMA) and single microspore stage (SCP) during pollen development in 02428-4x compared to 02428-2x, respectively (S12 Table). The Venn diagram results showed that 1421 DEGs (990 up- and 431 downregulated genes) were specifically detected in PMA (sDEGs-PMA), whereas 1927 DEGs (921 up- and 1006 downregulated) were specifically associated with SCP (sDEGs-SCP). Furthermore, 313 and 352 genes specifically showed upregulation and downregulation during meiosis (sDEGs-MA) in 02428-4x (S7 Fig; S13 Table).

Of the DEGs that occurred during pollen development in 02428-4x, 39 GO enrichment pathways were found, including those associated with the nucleolus, flower development, response to abiotic stimulus and carbohydrate metabolic process (S8 Fig). In total, eight prominent categories were associated with upregulated sDEGs-MA, such as response to stimulus and catalytic activity. In addition to upregulated sDEGs-MA, transcription factor activity was identified in downregulated sDEGs-MA (S9 Fig; S13 Table). A KEGG analysis indicated that the up- and downregulated DEGs during meiosis were enriched in five and eight pathways, respectively (S14 Table). Among the upregulated DEGs, eight were related to the plant hormone signal transduction pathway. In contrast, the downregulated genes were enriched in photosynthesis antenna proteins, porphyrin and chlorophyll metabolism, and photosynthesis pathways associated with photosynthesis metabolism in 02428-4x. Some important DEGs during meiosis were verified by qPCR, including five meiosis genes (*LOC_Os09g32020*, *LOC_Os03g58600*, *LOC_Os11g40150*, *LOC_Os02g37850* and *LOC_Os12g24140*), four meiosis stage-specific genes (*LOC_Os04g21590*, *LOC_Os01g68560*, *LOC_Os11g38620* and *LOC_Os11g38630*), two pollen sterility genes (*LOC_Os03g07250* and *LOC_Os08g03682*) and three tapetum genes (*LOC_Os06g40550*, *LOC_Os09g27620* and *LOC_Os10g35180*). The expression patterns of more than 90% (20/22) of the genes were similar to that of the transcriptome, demonstrating that the sequencing data is reliable (S10 Fig).

By comparing the miRNAome of pollen development between 02428-4x and 02428-2x [9], 158 DEGs were associated with differentially expressed miRNAs (DEM) in 02428-4x (S15 Table). MicroRNAs could modulate plant gene expression by gene silencing through inhibition of target mRNAs, so we focus on the negative regulation between miRNAs and genes. During PMA, 26 DEG-DEM pairs were found, 15 of these displayed negative regulation patterns, including *LOC_Os02g53830* (MTD1) and *LOC_Os04g01570* (invertase/pectin methylesterase inhibitor family protein), which were specifically downregulated during PMA in 02428-4x; additionally, their regulator *osa-miR164e_R-3* showed specific upregulation. A total of 37 DEG-DEM pairs were found in the SCP in 02428-4x. Of these, 22 exhibited negative regulatory patterns, including *LOC_Os04g59430* (*OsARF13*, auxin response factor), which showed a specific downregulation pattern and was targeted by the upregulated *osa-miR160a-5p_R-1_1ss20CT*. During MA, 26 DEG-DEM pairs were detected; among these, 14 showed negative regulatory patterns, including the specific downregulated genes, *LOC_Os08g23880* (no apical meristem protein) and *LOC_Os10g30150* (universal stress protein domain-containing protein), whose expressions were regulated by the upregulation of *gma-miR6300_R+3* and *osa-miR3979-5p_R+1*, respectively.

Among the DEGs in meiosis of 02428-4x, 446 depicted relationship with pollen fertility by comparing with pollen/meiosis/tapetum genes or meiosis stage-specific genes reported in rice, polyploid rice and *Arabidopsis* [7,37–41] (S16 Table). After comparing the DEGs to PMC-specific or meiosis stage-specific genes, 166 genes were found. Interestingly, 49 of 166 genes, which showed meiosis stage-specific expression in the report by Fujita et al. [38], generally displayed downregulation in 02428-4x compared to its diploid counterpart. Seven meiosis genes were found, and of those genes, *LOC_Os03g12414* (*OsSDS*, cyclin), *LOC_Os03g58600* (*MEL1*) and *LOC_Os11g40150* (*RAD51A1*, DNA repair protein Rad51) were upregulated in 02428-4x, whereas *LOC_Os09g32020* (*OsDFR*, ubiquitin fusion degradation protein) was downregulated in 02428-4x. In addition, five downregulated genes were associated with pollen sterility, including *LOC_Os08g03682* (*CYP703A3*, cytochrome P450) and *LOC_Os08g38810* (*RAFTIN*, BURP domain-containing protein), which showed specific expression patterns during meiosis in 02428-4x. Six tapetum genes were detected in the meiosis DEGs in 02428-4x; among them, five showed downregulation patterns, including *LOC_Os04g39470* (*OsMYB80*, MYB family transcription factor), *LOC_Os09g27620* (*PTC1*, PHD-finger domain-containing protein), *LOC_Os06g40550* (*OsABCG15*, ABC-2 type transporter domain-containing protein), *LOC_Os03g18480* (*OsTDF1*, MYB family transcription factor) and *LOC_Os10g35180* (*OsABCG26*, white-brown complex homolog protein 11). Furthermore, 87 (40 upregulated and 47 downregulated genes) DEGs were associated with the anther-related genes of neo-tetraploid rice (high fertility).

### Differentially methylated regions (DMRs) associated with meiosis in autotetraploid rice

During meiosis, the anthers of 02428-4x and 02428-2x were subjected to MeDIP-Seq to identify differentially methylated regions (DMRs), particularly those related to meiosis-related genes in 02428-4x. We obtained more than 20 million clean reads and the mapped reads were more than 95% (S11 Table). In total, 52484 regions displayed differential methylation between 02428-4x and 02428-2x, which were spread throughout the genome. Of these DMRs, 11367 and 41117 showed hypermethylation (high methylation levels) and hypomethylation (low methylation levels), respectively, in 02428-4x compared to 02428-2x (S17 Table). In total, 607 CpG islands (CGI) and 1475 CGI shores (~2 kb near the CpG islands) showed differential methylation in 02428-4x. Most of the CGI and CGI shores were enriched in transcription start sites (TSSs) and were hypermethylated, whereas higher numbers of intragenic CGI shores showed hypomethylation in 02428-4x (S11 Fig). Additionally, we further categorized the DMRs based on gene-body structure. Of the hypermethylated DMRs, 4170 were located in promoter regions that were more than the other regions in 02428-4x. In contrast, hypermethylated DMRs were spread in whole genome (promoter, exon, intron and intergenic), and promoter DMRs exhibited a little higher number than other regions (S11 Fig).

**Fig 5:**
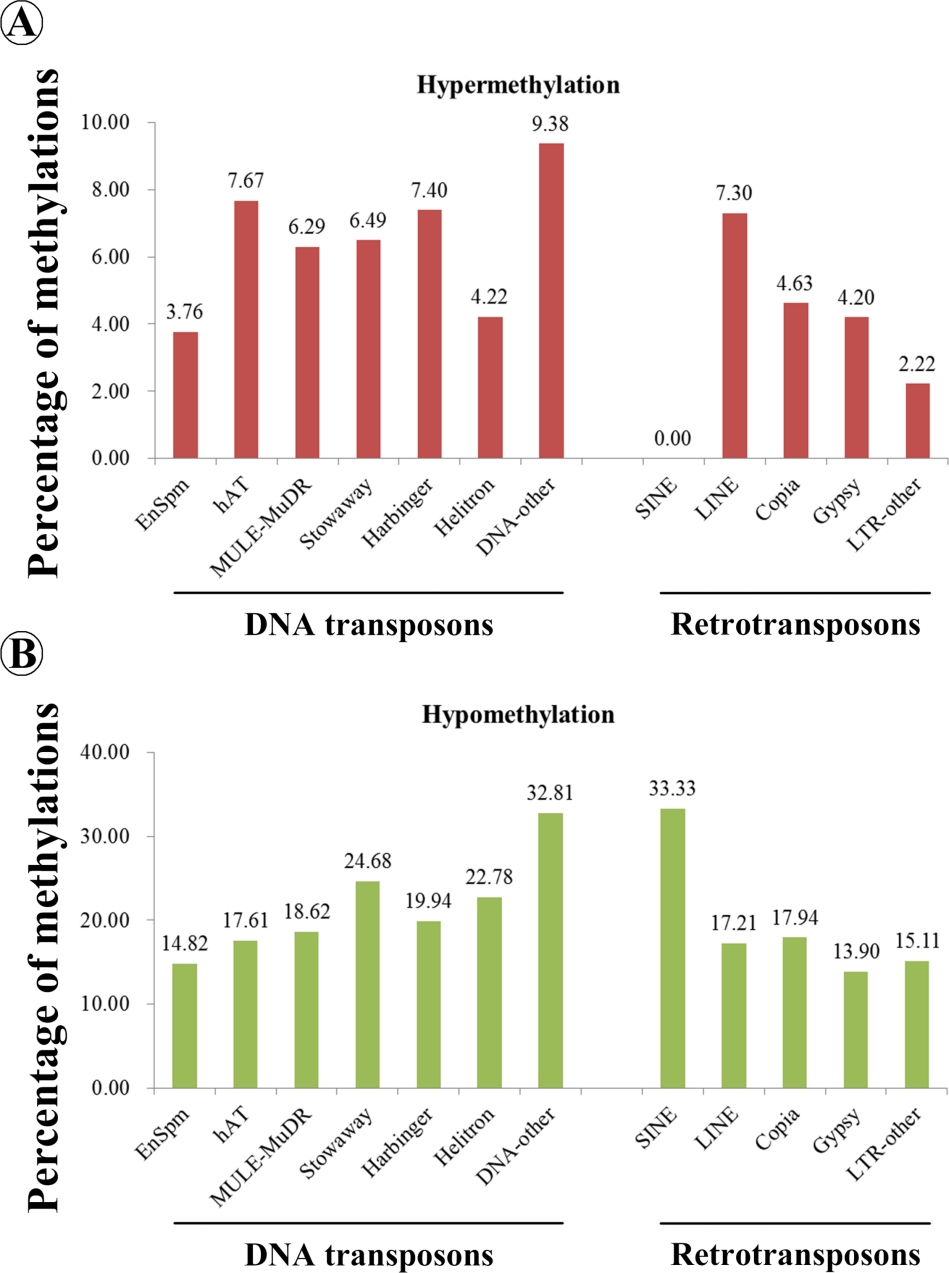
Distribution of methylated transposable element genes in 02428-4x.

Interestingly, 2870 DMRs related to repetitive elements (simple repeats and low complexity repeats) with 25.02% hypermethylation and 74.98% hypomethylation were identified in 02428-4x (S12 Fig). In addition, 3448 transposable element (TE) genes were corresponding to 9147 DMRs, including 1994 (21.39%) and 7153 (78.61%) hypermethylated and hypomethylated regions, respectively (S17 Table). Only 27 of 3448 TE genes showed homozygous SNPs/InDels between this resequencing data and previous dataset (2013). Based on the RepeatMasker annotation of whole-genome TEs, 22.34% of the DNA transposons (Class II) were associated with differential methylation in 02428-4x, and 19.21% of retrotransposons (Class I) were related to differential methylations. The hAT family of DNA transposons and LINE family of retrotransposons was dominant in the hypermethylation regions, whereas Stowaway family of DNA transposons and SINE family of retrotransposons showed a higher proportion in the hypomethylation regions (Fig 5).

A total of 13701 genes, corresponding to the 52484 DMRs, were detected. The expressions of genes were repressing/silencing by the DNA methylation in promoter and first exon regions [44,45]. In total, 1774 and 491 genes (2340 unigene) were associated with hypermethylation in promoter and first exon regions, respectively, whereas 11028 genes were hypomethylated on gene-body (promote, intron and exon) (S18 Table). Venn diagram showed that 475 genes were associated with differential methylation levels on different region in same gene, such as *LOC_Os03g44760*, which showed hypomethylation in promote region and displayed hypermethylation in exon region (S13 Fig). Meanwhile, 10553 and 1865 genes were specifically associated with hypomethylation and hypermethylation in 02428-4x, respectively (S13 Fig). Of the 10553 hypomethylated genes, 18 GO pathways were identified, including those related to the regulation of gene expression (epigenetic), reproduction and nucleotide binding (S14 Fig). 13 significant pathways were related to the hypomethylated genes, including 36 hypomethylated genes related to ribosome biogenesis in eukaryotes and 28 associated with nucleotide excision repair (S19 Table). No enrichment in GO and KEGG pathways was identified for the hypermethylated genes.

In total, 944 DMRs were found to be related to pollen/meiosis/tapetum genes and meiosis stage-specific genes by comparing with previous researches [7,37–41], 128 displayed hypermethylation, and 816 showed hypomethylation (S20 Table). Of the 128 hypermethylated genes, 43 were rice PMC-specific or meiosis stage-specific genes, such as *LOC_Os08g20200* (male sterility protein) which showed promoter hypermethylation. One tapetum gene, *LOC_Os12g36030* (*Pms3*, annotated as an expressed protein); and seven meiosis genes, including *LOC_Os02g04080* (*SMC3*, chromosome segregation protein sudA), *LOC_Os02g32570* (*RAD5a*, SNF2 family N-terminal domain-containing protein), *LOC_Os03g01590* (*PAIR1*), *LOC_Os06g04190* (*OsRAD1*, rad1), *LOC_Os07g16224* (*AGO9*, piwi domain-containing protein), *LOC_Os08g44350* (*AHP2*, histidine-containing phosphotransfer protein) and *LOC_Os09g35000* (*OsGEN1*, flap endonuclease), were found. In addition, 24 genes were related to anther of neo-tetraploid rice (high fertility), which showed hypermethylation in 02428-4x.

### Identification and functional annotation of low fertility-related genes with variations in autotetraploid rice

By combining the high-throughput sequencing results, we acquired multiple candidate genes showing variations during meiosis in autotetraploid rice. A total of 16 genes showed differential expressions and had sequence variations, but differential methylation regions were not found, and defined as Group I. 249 genes displayed differential expressions and differential methylations, but sequence variations were not detected, and defined as Group II. A total of 536 genes had differential methylation regions and sequence variations, but differential expressions were not identified, and defined as Group III (S21 Table). Additionally, combined with the DNA sequence variations, DNA methylation and DEGs, five genes (Group IV) were detected in 02428-4x. Six DEGs having CNVs in 02428-4x were found, of which, five showed positive relation between DEGs and CNVs, including four upregulated genes with gain CNVs and one downregulated gene with loss CNV (Table 2). 254 genes contained differential methylation regions, of which, 109 DEGs showed negative regulation with differential methylation, including 19 genes showed downregulation and hypermethylation, and 90 genes displayed upregulation and hypomethylation.

**Table 2:** Differentially expressed genes associated with copy number variations in autotetraploid rice.

Gene	Type	Annotation	Differential expression	Methylation	Sequence variations	Description	Reference
*LOC_Os07g07860*	Group I	LTPL76—Protease inhibitor/seed storage/LTP family protein precursor	Specific down-regulation		loss CNVs		
*LOC_Os01g04350*	Group I	hsp20/alpha crystallin family protein	Specific up-regulation		loss CNVs		
*LOC_Os11g44420*	Group I	expressed protein	Specific up-regulation		gain CNVs	meiosis stage-specific	Tang et al. 2010 [40]
*LOC_Os11g38630*	Group I	expressed protein	up-regulation		gain CNVs	meiosis stage-specific	Deveshwar et al. 2011 [37]
*LOC_Os11g38620*	Group IV	expressed protein	up-regulation	Hypomethylation (Promoter)	gain CNVs	meiosis stage-specific	Tang et al. 2010 [40]
*LOC_Os11g38640*	Group IV	expressed protein	up-regulation	Hypomethylation (Exon, Intron)	gain CNVs	anther-related in neo-tetraploid rice	Guo et al. 2017 [39]

Group I indicated the genes showed differential expressions and had sequence variations, but did not have differential methylation regions. Group IV indicated the genes showed DNA sequence variations, DNA methylation and differential expressions.

Of the Group I genes (16), five were related to pollen development, meiosis or tapetum and showed upregulation in meiosis in 02428-4x (S21 Table), including *LOC_Os11g03300* (NAC domain transcription factor) and *LOC_Os11g10590* (expressed protein) that upregulated during meiosis of Taichung65-4x [7]; *LOC_Os04g01690* (pyridoxal-dependent decarboxylase protein) and *LOC_Os11g37360* (an expressed protein) that the anther-related genes of high fertility neo-tetraploid rice [39]; *LOC_Os11g44420* (an expressed protein) and *LOC_Os11g38630* (an expressed protein) that the meiosis stage-specific genes [37,40].

Of the Group II genes (249), 98 were associated with pollen development, meiosis or tapetum (S21 Table). Three genes displayed downregulation and hypermethylation during meiosis in 02428-4x, including *LOC_Os02g05000* (expressed protein), which is specific in PMCs [40]; *LOC_Os10g33350* (endo-beta-N-acetylglucosaminidase) and *LOC_Os11g47840* (OsRhmbd18 putative rhomboid homolog) which were associated with anther in neo-tetraploid rice (high fertility) [39]. Furthermore, 38 genes were upregulated and hypomethylated in 02428-4x. Among these, 19 were related to upregulated genes in Taichung65-4x [7], and 14 were associated with PMC-specific or meiosis stage-specific expression patterns in rice [37.40], including *LOC_Os01g57610* (OsGH3.1, probable indole-3-acetic acid-amido synthetase) that targeted by *osa-miR1436_L+3_1ss5CT*. One gene, *LOC_Os06g08380* (GSL5, 1,3-beta-glucan synthase component domain-containing protein), which is related to male sterility, was also found in 02428-4x.

Of the Group III genes (536), only 17 were related to pollen development, meiosis or tapetum (S21 Table). Two tapetum genes, *LOC_Os05g47446* (*OsPDCD5*) showed hypomethylation and CNVs, and *LOC_Os10g03660* (*OsADF*) showed heterozygous SNPs/InDels (missense) and hypomethylation in 02428-4x. Nine genes were related to the rice PMC-specific and meiosis stage-specific expression patterns [38,40], such as *LOC_Os12g12260* (diacylglycerol kinase 1), which had homozygous SNPs/InDels in promoter, and displayed hypomethylation in exon and intron.

**Fig 6:**
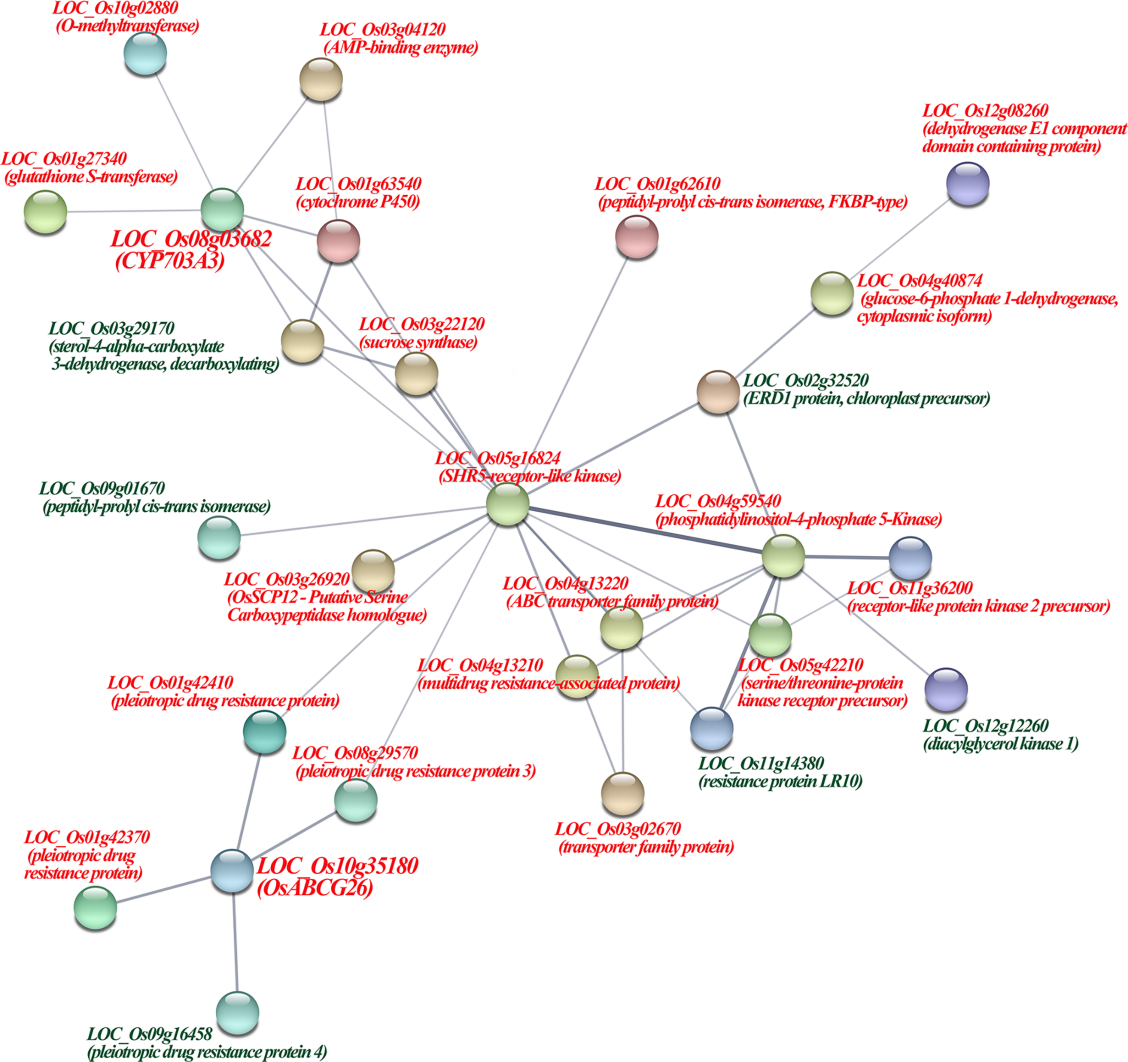
Protein-protein interactions of candidate meiosis-related genes in autotetraploid rice. Group 1 genes: genes related to DNA polymorphisms and that exhibit differential expression patterns. Group 2 genes: genes related to DNA methylation and that exhibit differential expression patterns. Group 3 genes: genes related to DNA polymorphisms and DNA methylation.

In addition, two genes related to meiosis were detected in the Group IV genes (5) by comparing with the previous researches [7,37–41] (S21 Table). *LOC_Os11g38640* (an expressed protein) showed upregulation, promoter hypomethylation and CNVs were related to the high fertility neo-tetraploid rice. *LOC_Os11g38620* (an expressed protein) that displayed upregulation, exon and intron hypomethylation and CNVs were associated with meiosis stage-specific expression. Additionally, the differential expression pattern of *LOC_Os11g38620* was confirmed via qPCR (S10 Fig). Taken together, 122 of 806 (Group I to IV) genes should be the low fertility-related genes (LFG) in autotetraploid rice. Moreover, 27 of these 122 LFG were involved in the protein-protein interaction network during meiosis in 02428-4x (Fig 6), including *LOC_Os10g35180* (*OsABCG26*) and *LOC_Os08g03682* (*CYP703A3*), which interacted with four and six genes, respectively.

## Discussion

### DNA sequence variations associated with meiosis might affect fertility in autotetraploid rice

In the present study, various types of polymorphic loci were found in autotetraploid rice compared with their diploid counterpart. These results suggested that next-generation sequencing technology can be a powerful tool for mining DNA polymorphisms [46,47], even in polyploid plants [48]. However, few homozygous SNPs/InDels (0.45%) were identified in autotetraploid rice compared to the diploids, and only eight genes were related to major impact mutations and missense mutations, which suggest that there are few homozygous SNPs/InDels between autotetraploid rice and diploid rice after autopolyploidization. In addition, these eight homozygous polymorphic genes did not show distinct functions in the meiosis of autotetraploid rice, demonstrating that they might play other roles in autotetraploid rice, except *LOC_Os09g37270* (*OsRacGEF1*). *OsRacGEF1* is involved in chitin-triggered immune responses and resistance to rice blast infection [49], but its role not yet reported in fertility. This gene, which was involved in megagametogenesis, showed missense mutation (arginine to histidine) in autotetraploid rice, and SNP was monitored by Sanger sequencing. Does this gene might affect the low fertility in autotetraploid rice? It requires further investigations. Moreover, most of the SNPs/InDels in autotetraploid rice were associated with heterozygosity. This phenomenon may be due to the four chromosomes in autotetraploid rice having heterozygous loci in 1:3 or 2:2 distributions. It is unknown whether these heterozygous loci can interact or not, regulate gene expression and play roles in autotetraploid rice; therefore, additional studies are needed. Beside, many low fertility-related genes associated with SVs/CNVs in autotetraploid rice. Five genes showed positive correlation with CNVs and qPCR results have revealed marked expression changes in these genes (i.e., *LOC_Os11g38620* and *LOC_Os11g38630*). The gained CNVs of these two meiosis stage-specific genes could change their differential expression levels in 02428-4x, which implied that the CNV might affect autotetraploid rice and cause undesirable traits. Therefore, we should focus on SVs/CNVs in follow-up studies of autotetraploid rice.

### The hypomethylation of DMR (differentially methylated regions) of autotetraploid rice might cause genome instability or abnormal gene transcription in anthers during meiosis

Zhang et al. [50] reported that the DNA methylation patterns were highly similar in three different ploidy (monoploid, diploid and triploid) rice lines, implying that ploidy may not have a widespread influence on DNA methylation. These findings were different from those in our study, in which ~80% of DMRs exhibited hypomethylation in autotetraploid rice compared to diploid rice. The hypomethylated DMRs were spread in every region (promoter, exon, intron and intergenic regions). Gene methylation serves to repress unintended transcription for efficient transcriptional elongation [51]. The hypomethylation in autotetraploid rice might be associated with unintended transcription initiation, which hinders the successful transcription of the gene. In *Arabidopsis*, *ROS1* is a major DNA demethylase that erase DNA methylation for gene transcriptional regulation [52]. We hypothesized that autotetraploid rice might have a gene that function as *ROS1* causing DNA hypomethylation after polyploidization. In addition, a high number of hypermethylated DMRs were located in the promoter regions, which demonstrated where the negative regulation of the gene transcribed starts and subsequently cause lower expression levels of some important genes in autotetraploid rice, such as low fertility-related genes. The relationship between the gene expression and methylation level was not linear in our study; 109 of 254 DEG-DMR pairs showed negative regulation. This result was consistent with the previous MeDIP-seq studies [15,16], such as Hu et al. [16] found that down-regulated genes did not show significant hypermethylation. The negative regulations of DEG-DMR pairs might due to other regulatory mechanisms, such as DNA hypermethylation of DNA methylation monitoring sequence (MEMS) in the promoter of the *ROS1* could concomitantly increase *ROS1* expression [53].

Furthermore, TEs associated with hypomethylation during meiosis in autotetraploid rice anthers were identified in the present study. Polyploidization could increase genetic mutations and changes in gene regulation due to the increases in TEs [54]. Hypomethylation of TEs may cause transposable element mobilization in young panicles at meiosis stage of PA64S (rice sterility line) [16]. Recently, we found that more than 80% of the differentially expressed 24 nt TEs-siRNAs (siRNAs associated with TEs) exhibited downregulation during pollen development of autotetraploid rice (same materials) and may activate TEs and induce genome destabilization [9]. These findings suggest that the irregular TEs associated with hypomethylation and downregulation of 24 nt TE-siRNAs results in an autotetraploid rice incompatibility response to “genomic shock” by polyploidization and probably disturbs chromatin structure during autotetraploid rice meiosis. However, we cannot detect the 3448 TEs genes’ expression levels in transcriptome. We speculated that the TEs have already triggered from these TE genes and transposed to other genes; or these TE genes were inactive in these materials. Zhang et al. [22] also described that the methylation level of TEs is different between autotetraploid rice panicles and those of their diploid counterpart; hypermethylated class II TEs have been reported in autotetraploid rice. However, these results are drastically different from our results. This difference is probably due to the different subspecies (*japonica* vs. *indica*) and tissues (anthers vs. panicles) in each experiment, and the WGD of rice needs to be further studied.

### The important genes related to DNA sequence variations, DNA methylations and differential expression patterns in autotetraploid rice might be co-associated with pollen sterility

In the present study, 122 genes were found to be related to low fertility in autotetraploid rice by comparing with the pollen/tapetum/meiosis-related genes and meiosis stage-specific genes [7,37–41]. Here, 31 of 122 genes showed meiosis stage-specific expressions, which played important role during pollen development of rice [37,38,40]. The abnormal changes of these genes might affect anther development in autotetraploid rice, such as *LOC_Os01g68560* (*OsMADS98*, MADS-box family gene with M-beta type-box) which displayed specific down-regulation and hypomethylation (Promoter) in autotetraploid rice. MADS-box genes play pivotal roles in the process of anther and pollen development [55]. MIKCc-type box genes (*OSMADS3* and *OSMADS58*) are crucial for regulating floral meristem determinacy [56], and dysfunctions of these genes cause severe abnormalities in floral meristem. MIKC*-Type MADS box genes (*MADS62*, *MADS63* and *MADS68*) required for pollen maturation [57], and knockdown or knockout lines showed severe defects in pollen maturation of rice. In addition, *OsMADS98* was also associated with the combined effect of polyploidy and pollen sterility loci interactions (IPE) and down-regulated in autotetraploid rice hybrid, which cause meiosis abnormalities and pollen sterility [41]. Here, the downregulation of *OsMADS98* in autotetraploid rice was confirmed by qPCR. These findings suggest that MADS-Box gene, *OsMADS98*, is important in autotetraploid rice, and its abnormal expression might hinder the procession of anther development in autotetraploid rice and cause male sterility.

Some important genes related to pollen sterility were also identified, including *LOC_Os08g03682* (*CYP703A3*), which was annotated as cytochrome P450 and was specifically downregulated and hypomethylated (Promoter), and *LOC_Os10g35180* (*OsABCG26*), which encodes white-brown complex homolog protein 11 and displayed downregulation and hypomethylation (Exon and Intron) during meiosis of autotetraploid rice. *CYP703A3* was highly expressed in the tapetum and weakly expressed in the microspores from stage 8 to stage 10 of anther development [58]. Although no apparent tapetum defect was found in the *cyp703a3* mutant, the product of *CYP703A3* serves as a backbone between the tapetum and pollen wall and is important for anther cuticle development and sporopollenin synthesis in rice. *OsABCG26* protein is localized on the plasma membrane of anther wall layers and is responsible for the transport of wax and cutin precursors from the tapetum to the anther surface [59]. Abnormal tapetal cells, persistent middle layers, collapsed microspores, and deeply stained debris in anther locules were identified in the *osabcg26* mutants. Two genes were involved in protein-protein interacted regulatory networks during meiosis in autotetraploid rice, and the downregulation of these genes was confirmed by qPCR. Moreover, the tapetum genes, *LOC_Os10g03660* (*OsADF*) [60] and *LOC_Os05g47446* (*OsPDCD5*) [61], also displayed DNA polymorphisms, DNA methylation or differential expression patterns in autotetraploid rice. Approximately 30% of abnormal tapeta were detected during autotetraploid rice anther development compared to diploid rice by semi-thin sections. These results mirrored the abovementioned differential expression pattern of the tapetum genes, which demonstrated that abnormal expression of tapetum genes might cause tapetum defects in autotetraploid rice. Moreover, *osa-miR2275* and 24 nt-phasiRNAs preferentially accumulate in the tapetum and meiocytes of maize [62]. Downregulated 24 nt-phasiRNAs showing similar spatial-temporal expression patterns as those of *osa-miR2275d* were found during meiosis in 02428-4x anthers [9], which implied that abnormal tapeta and tapetum genes might lead to irregular 24 nt-phasiRNAs and aggravate pollen sterility in autotetraploid rice.

Moreover, *LOC_Os11g38620* and *LOC_Os11g38630* combined with gain CNVs exhibited differential expression patterns in autotetraploid rice. Even though the function of these genes is largely unknown, they showed stage-specific expression during meiosis in rice. The function of these gene related to low fertility of autotetraploid rice should be further investigated. 122 genes could be elite male sterility genes that could be used for further studies about autotetraploid rice. However, the changes in gene expression between methylation levels and DNA polymorphisms are different and complex in autotetraploid rice. These changes might be regulated by another mechanism or regulators, such as long noncoding RNAs or circular RNAs, to respond to polyploidy effects.

**Fig 7:**
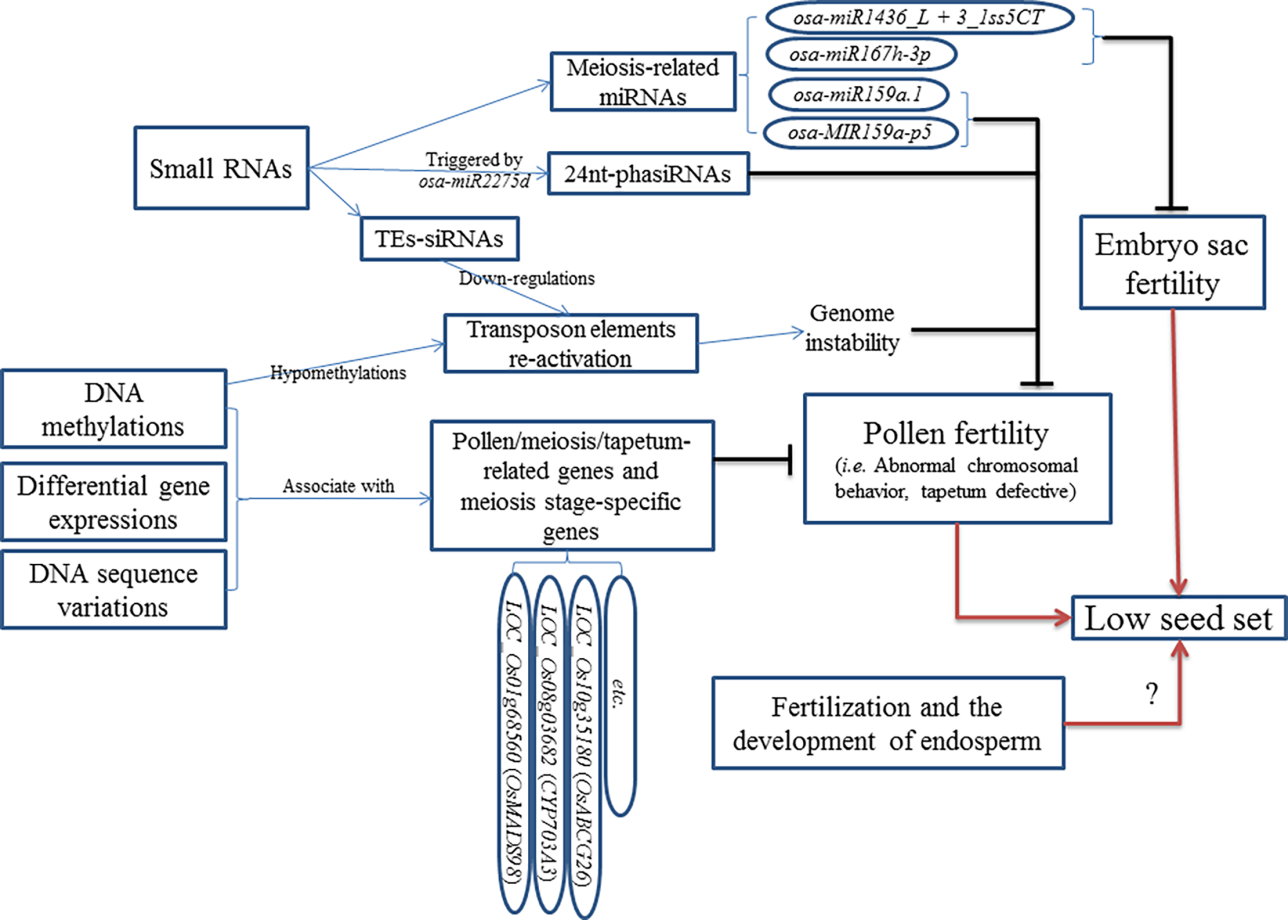
A hypothesis model of low seed set in autotetraploid rice. Blue line indicated the action for the next step. Black line means it cause bad effect to pollen and embryo sac fertility. Red line indicated that the low seed set may cause by multiple factors.

Overall, a low seed set (~15%) as well as poor pollen fertility (~43%) and abnormal chromosomal behavior (~20%) in 02428-4x was reported in our previous study [9]. These phenomena represent one reason for the low fertility caused by cytogenetics. Furthermore, abnormal tapeta (~30%) detected during anther development in 02428-4x further intensified pollen sterility. Various pollen/meiosis/tapetum-related genes and meiosis stage-specific genes were associated with different regulatory mechanisms; some showed DNA variation, some displayed differential expression, and some exhibited methylation in autotetraploid rice, even regulated by small RNAs [8,9]. Abnormal variations/expressions/methylations of low fertility-related genes cause sterile phenotype in autotetraploid rice. Taken together, these results showed that the low fertility in autotetraploid rice might be cause by multiple factors simultaneously or successively. Additionally, whether fertilization and endosperm development are normal or not and the sterility of autotetraploid rice should be further studied (Fig 7).

## Supporting information

S1 Fig Semi-thin section of anther development in 02428-2x. (A) pre-meiotic interphase, (B-D) meiosis stage, (E-G) single microspore stage, (H, I) mature pollen. Ep, En, ML and Ta indicate epidermis, endothecium, middle layer and tapetum. Bars = 50 μm.
(DOCX)

S2 Fig **Summary of variations in 02428-2x (A) and 02428-4x (B) compared to Nipponbare reference genome.** The chromosomes are presented along the perimeter of each circle. The circle from outside to inside indicate the coordinate of chromosome for SNP density, InDel density, CNV density and SV density (INS, DEL, INV, ITX (red line) and CTX (green line)); the distribution unit of genome is 1Mb. Circles were drawn by the Circos platform.
(DOCX)

S3 Fig Summary of the SNPs and InDels in 02428-4x and 02428-2x. (A, B) Change rate of SNPs (A) and InDels (B) in each chromosome. Change rate = chromosome length (bp)/variants number. The y-axis represents the change rate (bp). (C) Distribution of the transitions and transversions in SNPs. The y-axis represents the percentage of transitions/transversions number. (D) Distribution of the length of InDels. The y-axis represents the number of InDels at each length.
(DOCX)

S4 Fig Distribution of the polymorphic loci (SNPs & InDels) in 02428-4x compared to 02428-2x. The y-axis represents the number of SNPs and InDels in per 100kb of chromosome.
(DOCX)

S5 Fig Classification of the structural variations (SVs) (A) and copy number variations (CNVs) (B) in 02428-4x compared to 02428-2x.
(DOCX)

S6 Fig Classification of the DNA polymorphic genes in 02428-4x. (DOCX)

S7 Fig Classification of the differentially expressed genes during pollen development in 02428-4x. (DOCX)

S8 Fig Gene Ontology analysis of the differentially expressed genes (DEGs) during pollen development in 02428-4x. (A) Biological process category, (B) Molecular function category, (C) Cellular component category. Arrows and shading are defined in the key.
(DOCX)

S9 Fig Gene Ontology analysis of the differentially expressed genes (DEGs) during meiosis of 02428-4x. Arrows and shading are defined in the key in S8 Fig.
(DOCX)

S10 Fig qPCR verification of the differentially expressed genes (DEGs) during meiosis in 02428-4x. The x- and y-axis represent the DEGs and relative expression levels, respectively. Error bars represent the standard deviation (SD) of three biological replicates.
(DOCX)

S11 Fig Distribution of the differentially methylated regions during meiosis in 02428-4x. (A, B) Hypermethylation (A) and hypomethylation (B) of CpG Islands and CpG Islands shores. TSS (transcription start site): CGI/CGI shores located in 1000bp upstream to 300 downstream of TSS. TES (transcription end site): CGI/CGI shores located in 300bp upstream to 300 downstream of TES. Intragenic: CGI/CGI shores located in 300bp downstream of TSS to 300bp upsteam of TES. Intergenic: CGI/CGI shores located in 300bp downstream of TES to 1000bp upsteam of next gene TES. (C, D) Distribution of the hypermethylated (C) and hypomethylated (D) regions base on gene-body structure.
(DOCX)

S12 Fig DMRs associated with repetitive elements in 02428-4x. (DOCX)

S13 Fig Venn analysis of the hypomethylated and hypermethylated genes in 02428-4x. (DOCX)

S14 Fig Gene Ontology analysis of the hypomethylated genes during meiosis in 02428-4x. Arrows and shading are defined in the key in S8 Fig.
(DOCX)

S1 Table PCR verification of the polymorphic loci in 02428-2x and 02428-4x. (XLSX)

S2 Table The qPCR primers used in the present study. (XLSX)

S3 Table Summarization of semi-thin section of the anther development in 02428-4x and 02428-2x. (XLSX)

S4 Table Summary of the resequencing data of 02428-4x and 02428-2x mapped onto the reference genome. (XLSX)

S5 Table Summary of the sequence variations detected in 02428-4x and 02428-2x. (XLSX)

S6 Table The polymorphic loci (SNPs & InDels) between 02428-4x and 02428-2x. (XLSX)

S7 Table Position and annotation of the polymorphic loci (SNPs & InDels) in 02428-4x. (XLSX)

S8 Table The DNA homozygous and heterozygous polymorphisms between 02428-4x and 02428-2x. (XLSX)

S9 Table A list of DNA sequence variations in 02428-4x. (XLSX)

S10 Table DNA polymorphic genes associated with meiosis in 02428-4x. (XLSX)

S11 Table Overview of reads in diploid and autotetraploid rice during pollen development. (XLSX)

S12 Table A list of differentially expressed genes during pollen development in autotetraploid rice. (XLSX)

S13 Table Summary of differentially expressed genes (DEGs) in 02428-4x compared to 02428-2x. (XLSX)

S14 Table KEGG of the differentially expressed genes (DEGs) during meiosis in 02428-4x. (XLSX)

S15 Table Differentially expressed genes (DEG) associated with differentially expressed miRNAs (DEM) during pollen development in 02428-4x. (XLSX)

S16 Table The differentially expressed genes associated with meiosis in 02428-4x. (XLSX)

S17 Table Summary of the differentially methylated regions during meiosis in 02428-4x. (XLSX)

S18 Table A list of gene associated with differential methylated region. (XLSX)

S19 Table KEGG of the hypomethylated genes during meiosis in 02428-4x. (XLSX)

S20 Table The differentially methylated genes associated with meiosis in 02428-4x. (XLSX)

S21 Table The candidate genes associated with low fertility in autotetraploid rice. (XLSX)

## Abbreviations

CNVs: copy number variations
DEGs: differentially expressed genes
DMRs: differentially methylated regions
InDels: insertion–deletion polymorphisms
MA: meiosis
SNPs: single nucleotide polymorphisms
SVs: structural variations
TEs: transposable elements
