# Supplementary material for: Genome-wide analysis of DNA polymorphisms, the methylome and transcriptome revealed that multiple factors are associated with low pollen fertility in autotetraploid rice
Source: PLoS One. 2018 Aug 6;13(8):e0201854. doi: 10.1371/journal.pone.0201854 (PMC6078310; doi:10.1371/journal.pone.0201854)
Supplement: S1 Fig — (A) pre-meiotic interphase, (B-D) meiosis stage, (E-G) single microspore stage, (H, I) mature pollen. Ep, En, ML and Ta indicate epidermis, endothecium, middle layer and tapetum. Bars = 50 μm. (DOCX) [file pone.0201854.s001.docx]

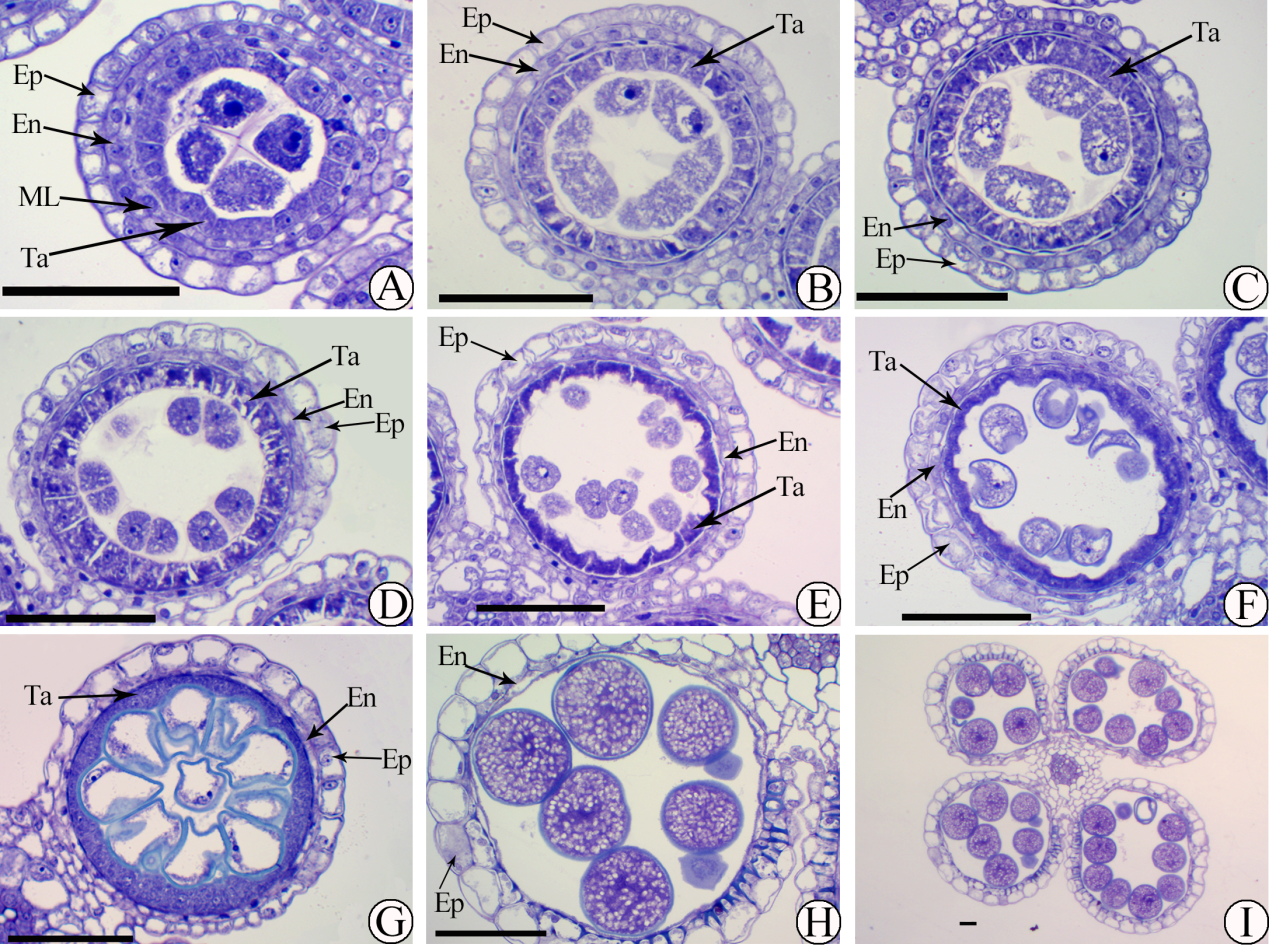


**S1 Fig. Semithin section of anther development in 02428-2x.** (A) pre-meiotic interphase, (B-D) meiosis stage, (E-G) single microspore stage, (H, I) mature pollen. Ep, En, ML and Ta indicate epidermis, endothecium, middle layer and tapetum. Bars=50 μm.
