# Supplementary material for: Genome-wide analysis of DNA polymorphisms, the methylome and transcriptome revealed that multiple factors are associated with low pollen fertility in autotetraploid rice
Source: PLoS One. 2018 Aug 6;13(8):e0201854. doi: 10.1371/journal.pone.0201854 (PMC6078310; doi:10.1371/journal.pone.0201854)
Supplement: S2 Fig — Summary of variations in 02428-2x (A) and 02428-4x (B) compared to Nipponbare reference genome. The chromosomes are presented along the perimeter of each circle. The circle from outside to inside indicate the coordinate of chromosome for SNP density, InDel density, CNV density and SV density (INS, DEL, INV, ITX (red line) and CTX (green line)); the distribution unit of genome is 1Mb. Circles were drawn by the Circos platform. (DOCX) [file pone.0201854.s002.docx]

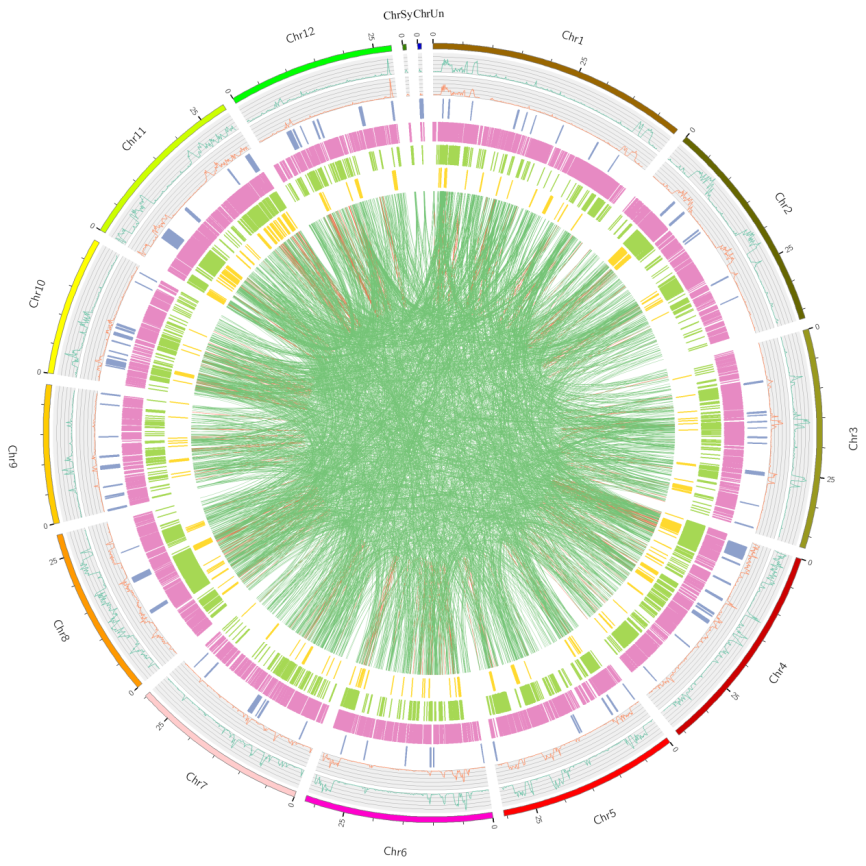


A


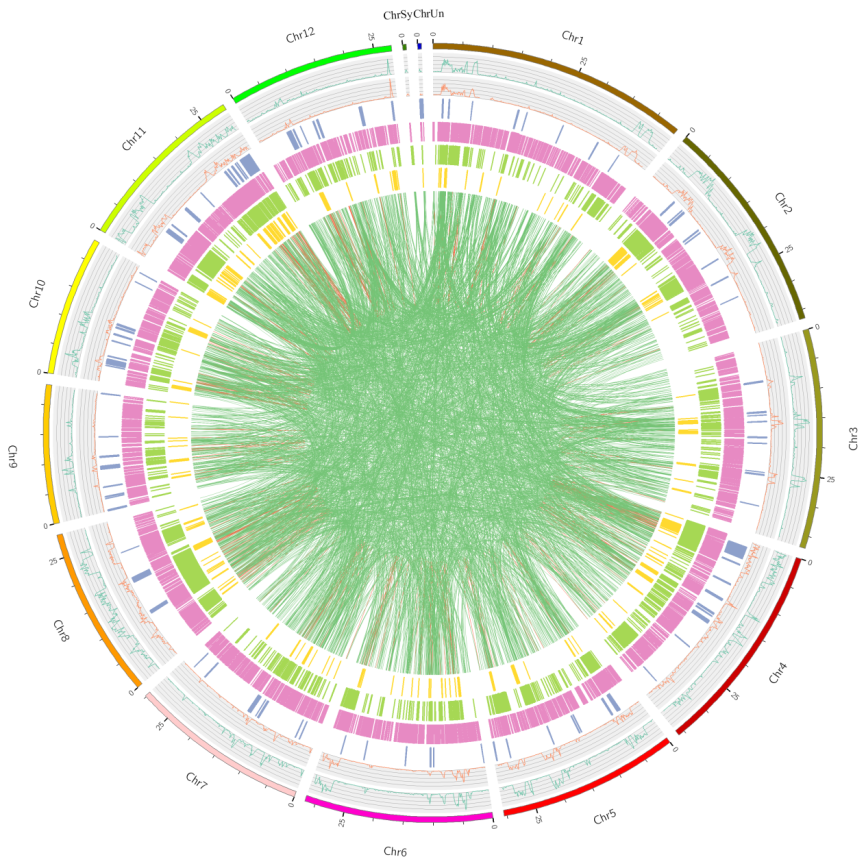


B

**S2 Fig. Summary of variations in 02428-2x (A) and 02428-4x (B) compared to Nipponbare reference genome.** The chromosomes are presented along the perimeter of each circle. The circle from outside to inside indicate the coordinate of chromosome for SNP density, InDel density, CNV density and SV density (INS, DEL, INV, ITX (red line) and CTX (green line)); the distribution unit of genome is 1Mb. Circles were drawn by the Circos platform.
