# Supplementary material for: Genome-wide analysis of DNA polymorphisms, the methylome and transcriptome revealed that multiple factors are associated with low pollen fertility in autotetraploid rice
Source: PLoS One. 2018 Aug 6;13(8):e0201854. doi: 10.1371/journal.pone.0201854 (PMC6078310; doi:10.1371/journal.pone.0201854)
Supplement: S3 Fig — (A, B) Change rate of SNPs (A) and InDels (B) in each chromosome. Change rate = chromosome length (bp)/variants number. The y-axis represents the change rate (bp). (C) Distribution of the transitions and transversions in SNPs. The y-axis represents the percentage of transitions/transversions number. (D) Distribution of the length of InDels. The y-axis represents the number of InDels at each length. (DOCX) [file pone.0201854.s003.docx]

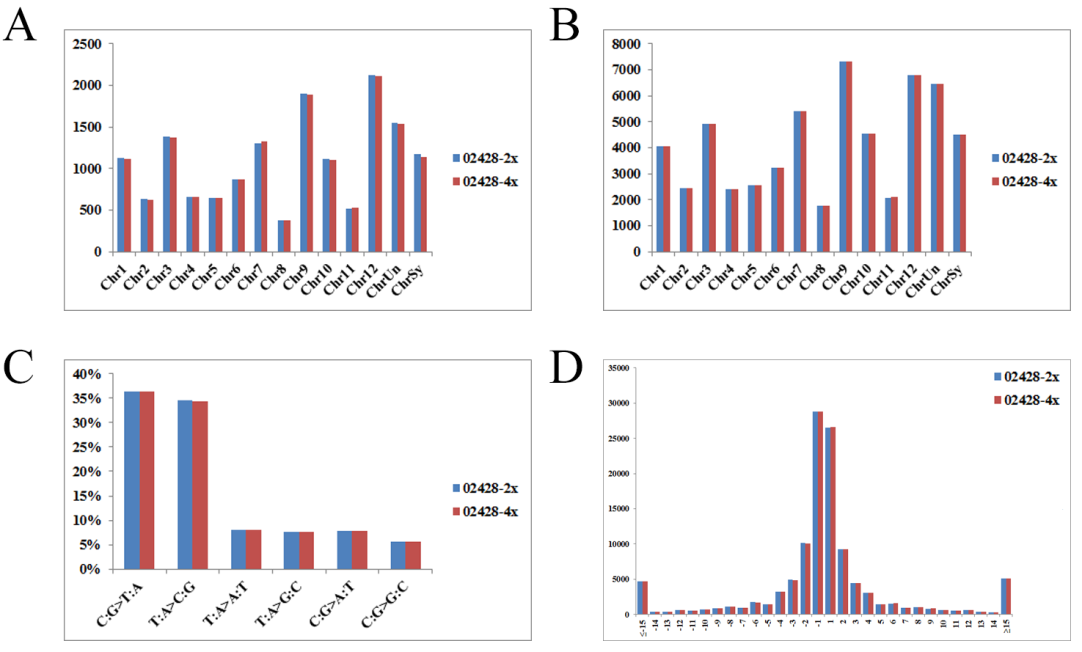


**S3 Fig. Summary of the SNPs and InDels in 02428-4x and 02428-2x.** (A, B) Change rate of SNPs (A) and InDels (B) in each chromosome. Change rate = chromosome length (bp)/variants number. The y-axis represents the change rate (bp). (C) Distribution of the transitions and transversions in SNPs. The y-axis represents the percentage of transitions/transversions number. (D) Distribution of the length of InDels. The y-axis represents the number of InDels at each length.
