# Supplementary material for: Genome-wide analysis of DNA polymorphisms, the methylome and transcriptome revealed that multiple factors are associated with low pollen fertility in autotetraploid rice
Source: PLoS One. 2018 Aug 6;13(8):e0201854. doi: 10.1371/journal.pone.0201854 (PMC6078310; doi:10.1371/journal.pone.0201854)
Supplement: S4 Fig — The y-axis represents the number of SNPs and InDels in per 100kb of chromosome. (DOCX) [file pone.0201854.s004.docx]

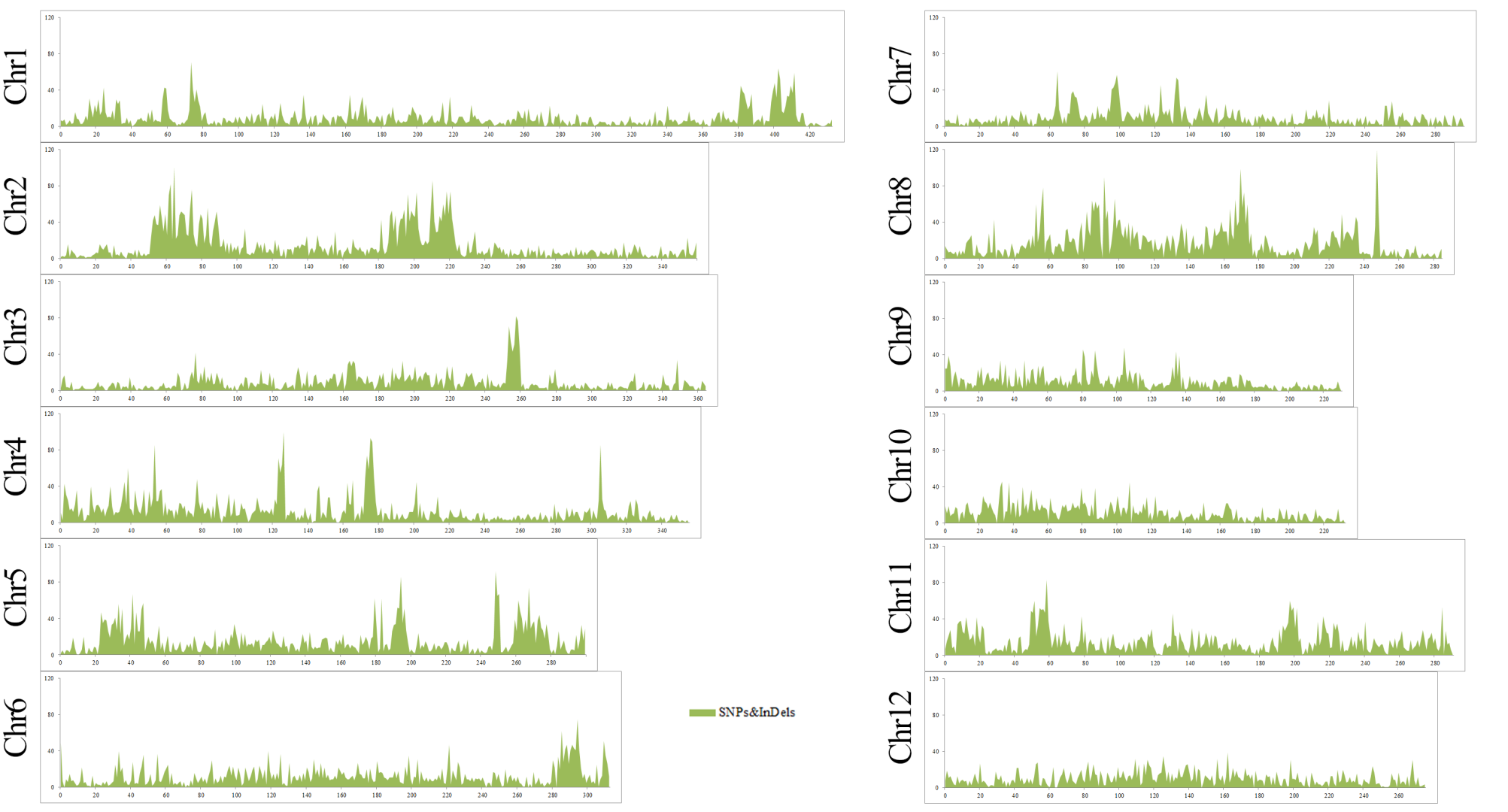


**S4 Fig. Distribution of the polymorphic loci (SNPs & InDels) in 02428-4x compared to 02428-2x.** The y-axis represents the number of SNPs and InDels in per 100kb of chromosome.
