# Supplementary material for: Genome-wide analysis of DNA polymorphisms, the methylome and transcriptome revealed that multiple factors are associated with low pollen fertility in autotetraploid rice
Source: PLoS One. 2018 Aug 6;13(8):e0201854. doi: 10.1371/journal.pone.0201854 (PMC6078310; doi:10.1371/journal.pone.0201854)
Supplement: S5 Fig — Classification of the structural variations (SVs) (A) and copy number variations (CNVs) (B) in 02428-4x compared to 02428-2x. (DOCX) [file pone.0201854.s005.docx]

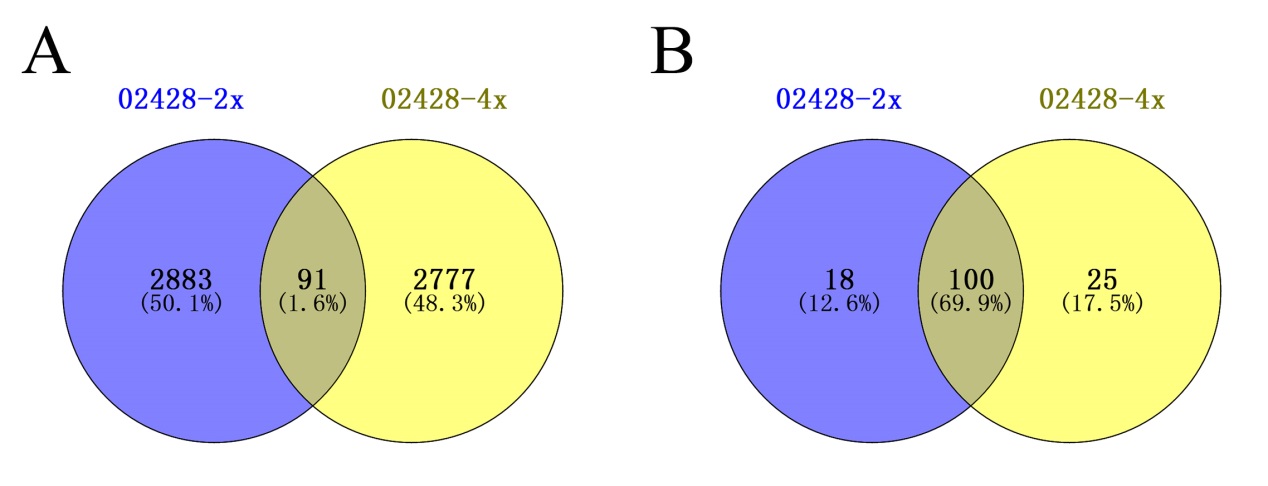


**S5 Fig. Classification of the structural variations (SVs) (A) and copy number variations (CNVs) (B) in 02428-4x compared to 02428-2x.**
