# Supplementary figures and images for: Genome-wide analysis of DNA polymorphisms, the methylome and transcriptome revealed that multiple factors are associated with low pollen fertility in autotetraploid rice
Source: PLoS One. 2018 Aug 6;13(8):e0201854. doi: 10.1371/journal.pone.0201854 (PMC6078310; doi:10.1371/journal.pone.0201854)

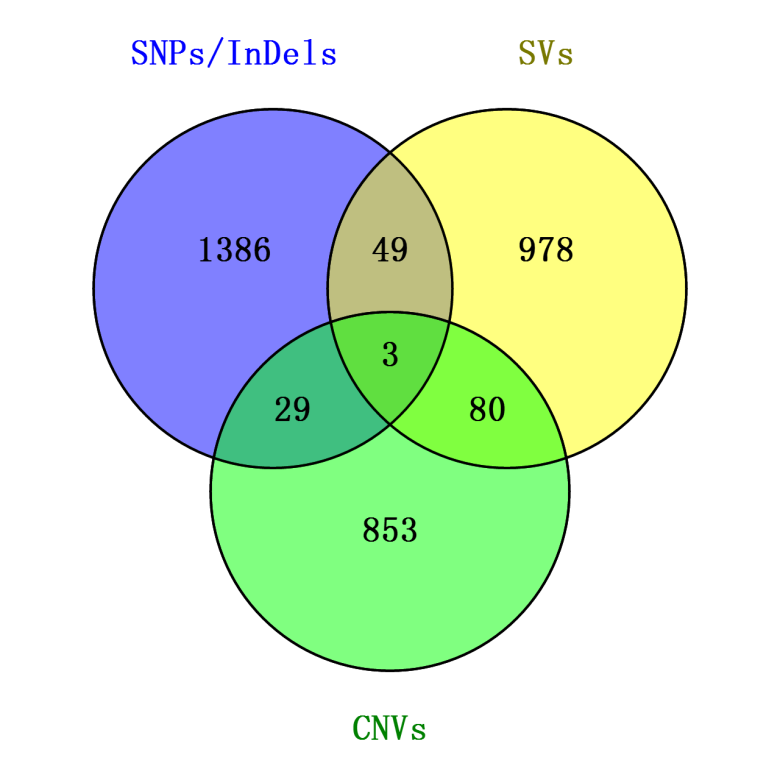


**S6 Fig. Classification of the DNA polymorphic genes in 02428-4x.**

Supplement: S6 Fig — (DOCX) [file pone.0201854.s006.docx]

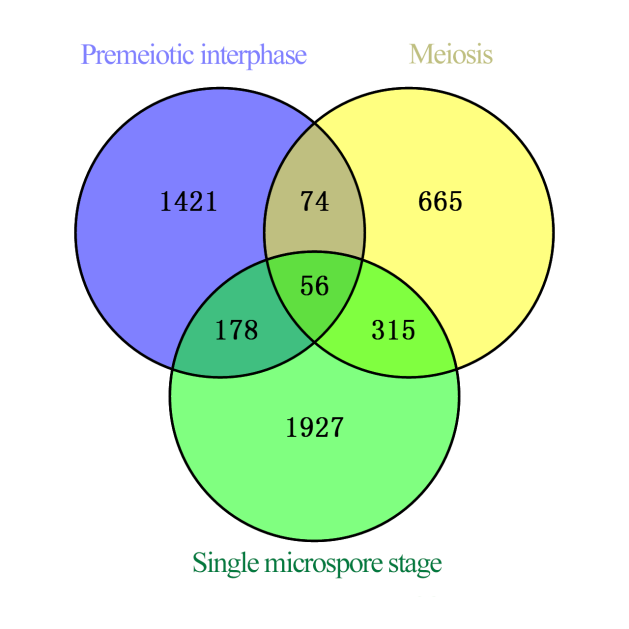


**S7 Fig. Classification of the differentially expressed genes during pollen development in 02428-4x.**

Supplement: S7 Fig — (DOCX) [file pone.0201854.s007.docx]

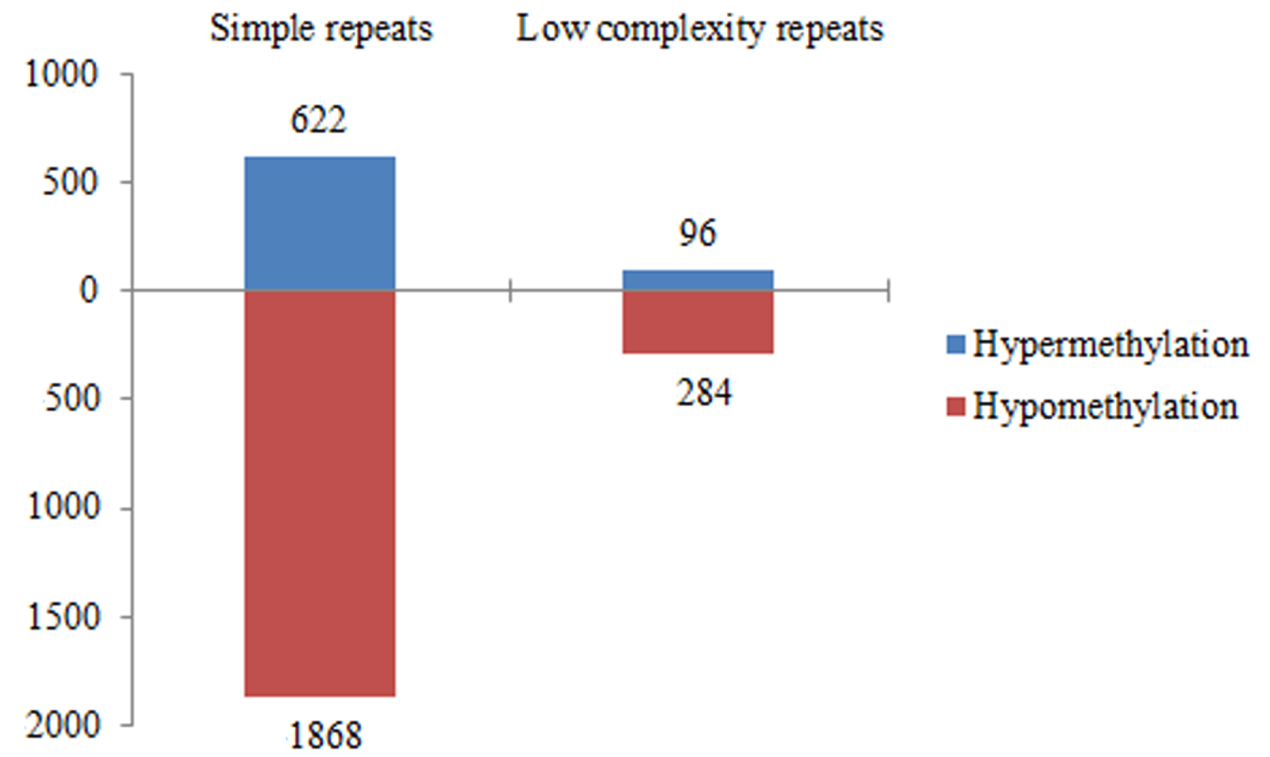


**S12 Fig. DMRs associated with repetitive elements in 02428-4x.**

Supplement: S12 Fig — (DOCX) [file pone.0201854.s012.docx]

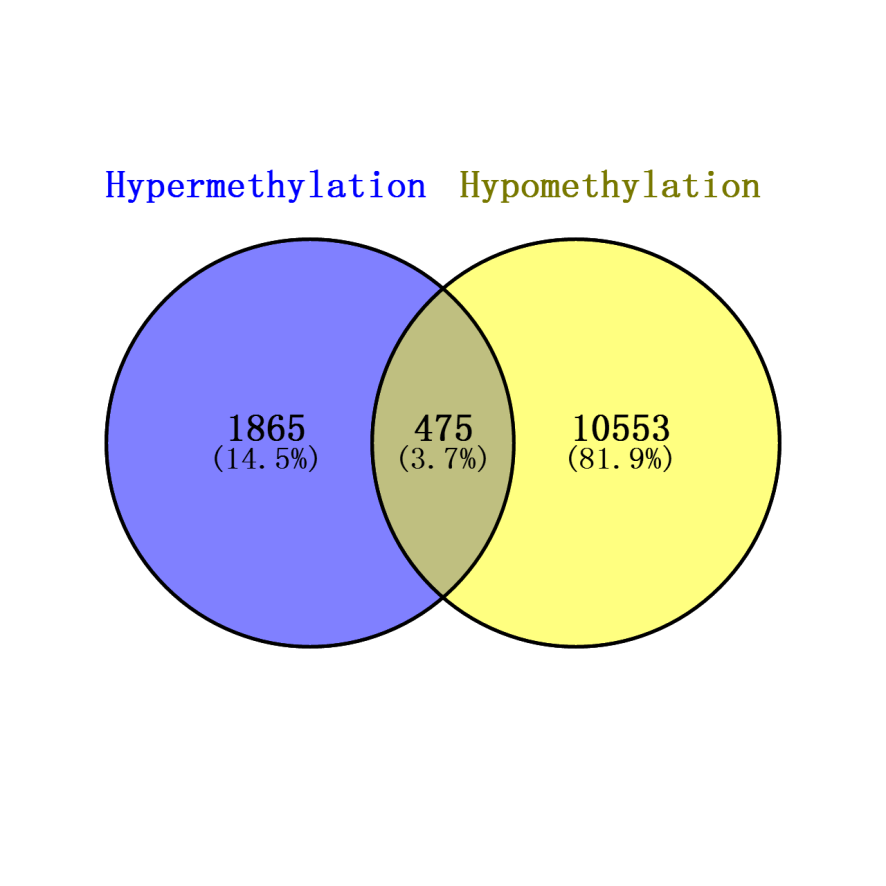


**S13 Fig. Venn analysis of the hypomethylated and hypermethylated genes in 02428-4x.**

Supplement: S13 Fig — (DOCX) [file pone.0201854.s013.docx]
