# Supplementary material for: Genome-wide analysis of DNA polymorphisms, the methylome and transcriptome revealed that multiple factors are associated with low pollen fertility in autotetraploid rice
Source: PLoS One. 2018 Aug 6;13(8):e0201854. doi: 10.1371/journal.pone.0201854 (PMC6078310; doi:10.1371/journal.pone.0201854)
Supplement: S10 Fig — The x- and y-axis represent the DEGs and relative expression levels, respectively. Error bars represent the standard deviation (SD) of three biological replicates. (DOCX) [file pone.0201854.s010.docx]

**S10 Fig. qPCR verification of the differentially expressed genes (DEGs) during meiosis in 02428-4x.** The x- and y-axis represent the DEGs and relative expression levels, respectively. Error bars represent the standard deviation (SD) of three biological replicates.
