# Supplementary material for: Genome-wide analysis of DNA polymorphisms, the methylome and transcriptome revealed that multiple factors are associated with low pollen fertility in autotetraploid rice
Source: PLoS One. 2018 Aug 6;13(8):e0201854. doi: 10.1371/journal.pone.0201854 (PMC6078310; doi:10.1371/journal.pone.0201854)
Supplement: S11 Fig — (A, B) Hypermethylation (A) and hypomethylation (B) of CpG Islands and CpG Islands shores. TSS (transcription start site): CGI/CGI shores located in 1000bp upstream to 300 downstream of TSS. TES (transcription end site): CGI/CGI shores located in 300bp upstream to 300 downstream of TES. Intragenic: CGI/CGI shores located in 300bp downstream of TSS to 300bp upsteam of TES. Intergenic: CGI/CGI shores located in 300bp downstream of TES to 1000bp upsteam of next gene TES. (C, D) Distribution of the hypermethylated (C) and hypomethylated (D) regions base on gene-body structure. (DOCX) [file pone.0201854.s011.docx]

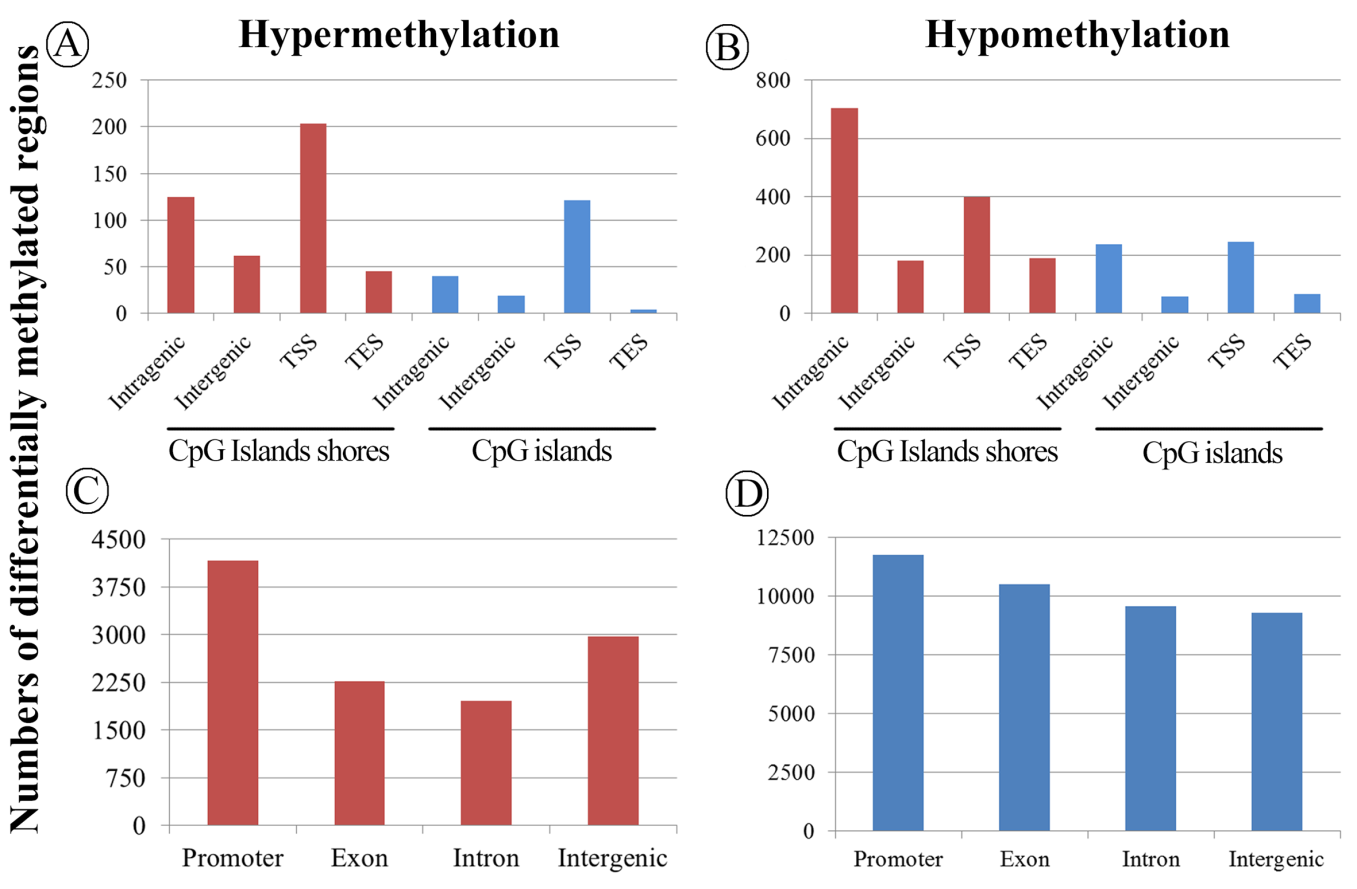


**S11 Fig. Distribution of the differentially methylated regions during meiosis in 02428-4x.** (A, B) Hypermethylation (A) and hypomethylation (B) of CpG Islands and CpG Islands shores. TSS (transcription start site): CGI/CGI shores located in 1000bp upstream to 300 downstream of TSS. TES (transcription end site): CGI/CGI shores located in 300bp upstream to 300 downstream of TES. Intragenic: CGI/CGI shores located in 300bp downstream of TSS to 300bp upsteam of TES. Intergenic: CGI/CGI shores located in 300bp downstream of TES to 1000bp upsteam of next gene TES. (C, D) Distribution of the hypermethylated (C) and hypomethylated (D) regions base on gene-body structure.
