# Supplementary material for: Genome-wide analysis of DNA polymorphisms, the methylome and transcriptome revealed that multiple factors are associated with low pollen fertility in autotetraploid rice
Source: PLoS One. 2018 Aug 6;13(8):e0201854. doi: 10.1371/journal.pone.0201854 (PMC6078310; doi:10.1371/journal.pone.0201854)
Supplement: S14 Fig — Arrows and shading are defined in the key in S8 Fig. (DOCX) [file pone.0201854.s014.docx]

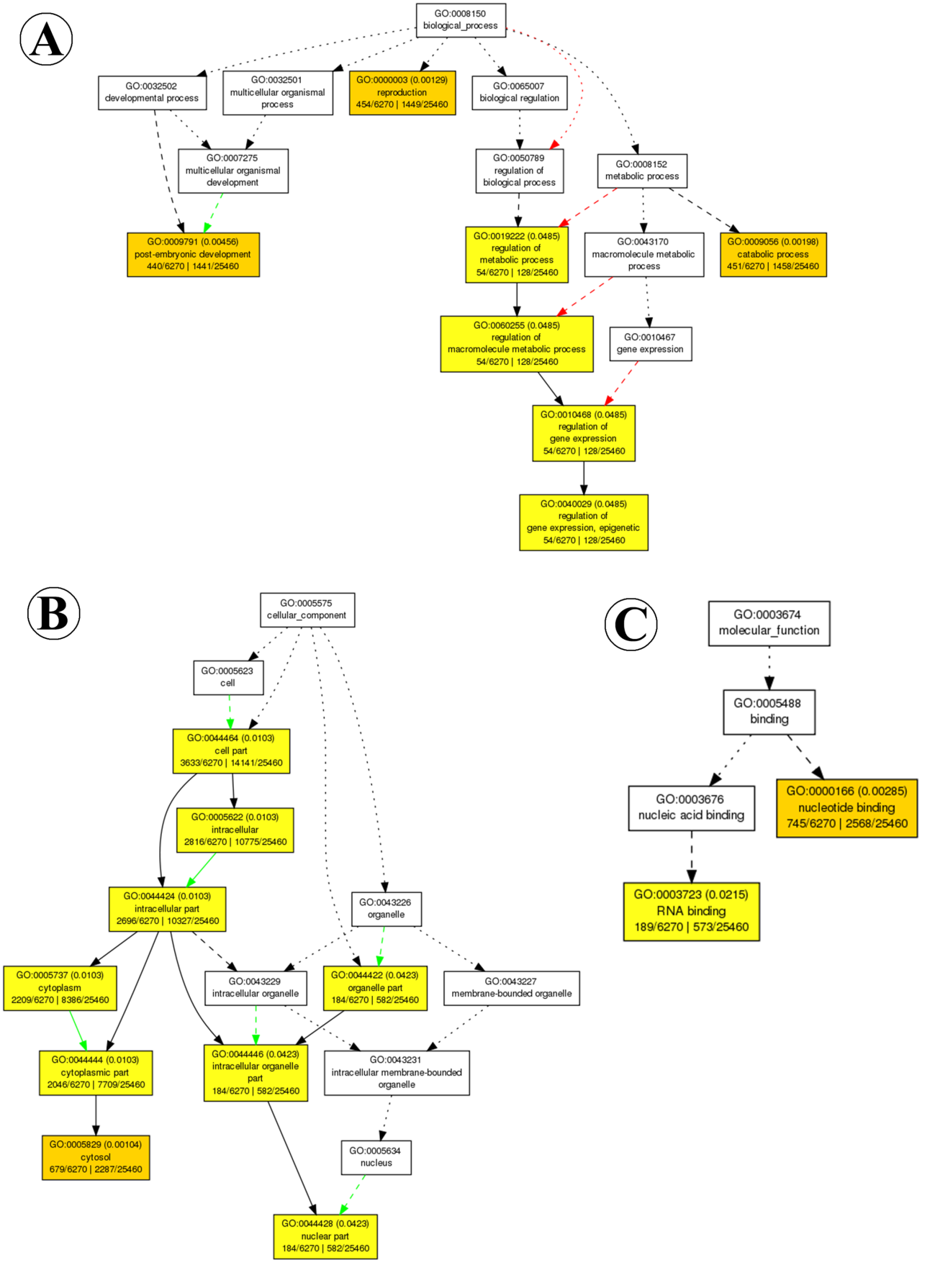


**S14 Fig. Gene Ontology analysis of the hypomethylated genes during meiosis in 02428-4x.** Arrows and shading are defined in the key in S8 Fig.
